# Supplementary figures and images for: Endophyte genomes support greater metabolic gene cluster diversity compared with non-endophytes in Trichoderma
Source: PLoS One. 2023 Dec 21;18(12):e0289280. doi: 10.1371/journal.pone.0289280 (PMC10735191; doi:10.1371/journal.pone.0289280)

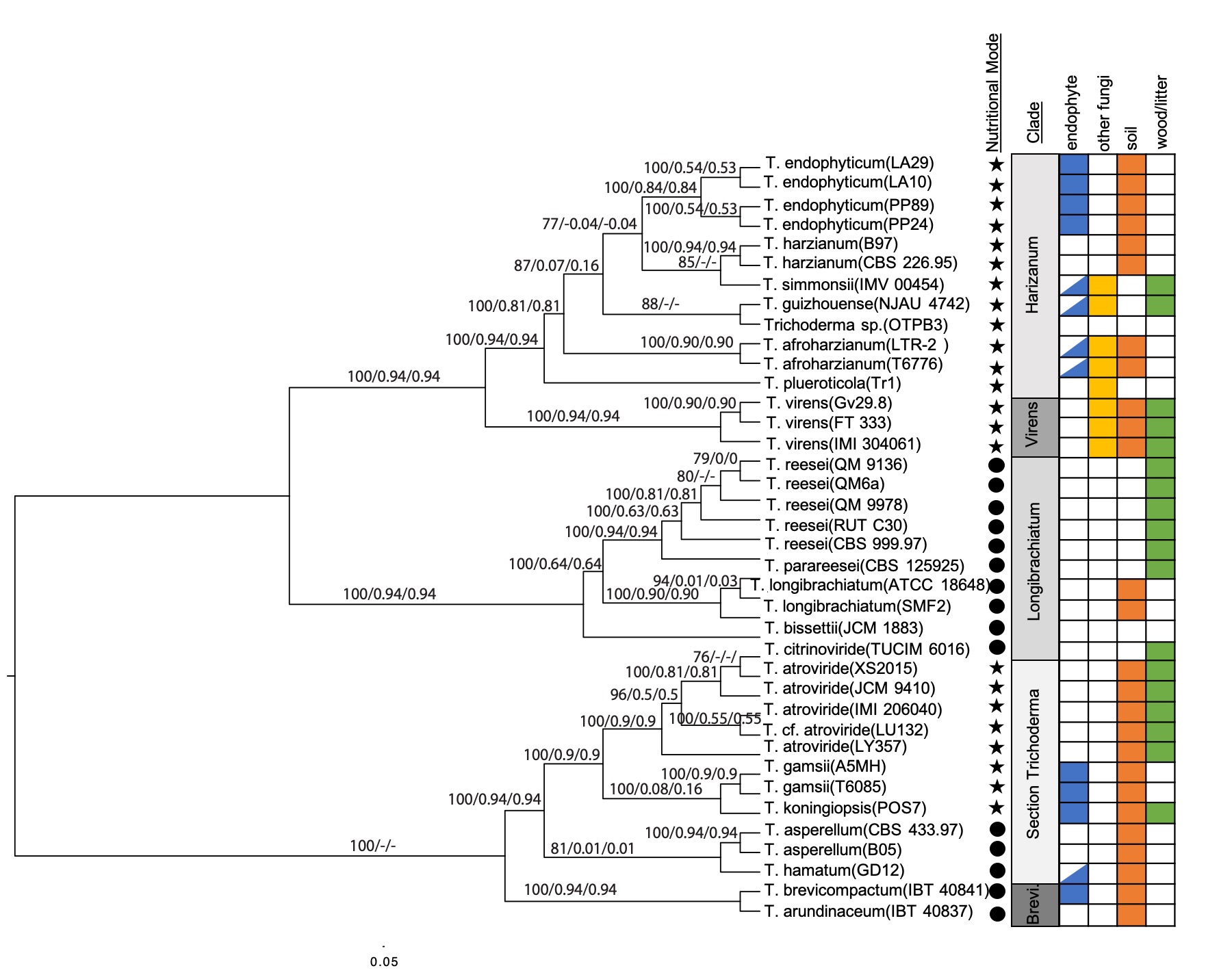

Supplement: S1 Fig — This consensus phylogenomic tree was created from 154 SCO IQ-TREEs which had >98% BP, using 10,000 bootstraps. Values at nodes represent the IQ-TREE maximum likelihood BP, Internode Certainty (IC), and Internode Certainty All (ICA) (BP/IC/ICA). Internode Certainty (IC) and Internode Certainty All (ICA) values were obtained from the corresponding RAxML majority rule extended consensus tree. Species lifestyle is indicated by presence/absence of color-coordinated cell, nutritional mode is indicated by either a circle (saprotroph) or a star (mycotroph). Endophyte cells half-filled indicated a potential endophytic lifestyle for that species. Branch lengths are not proportional to sequence divergence. (JPG) [file pone.0289280.s001.jpg]

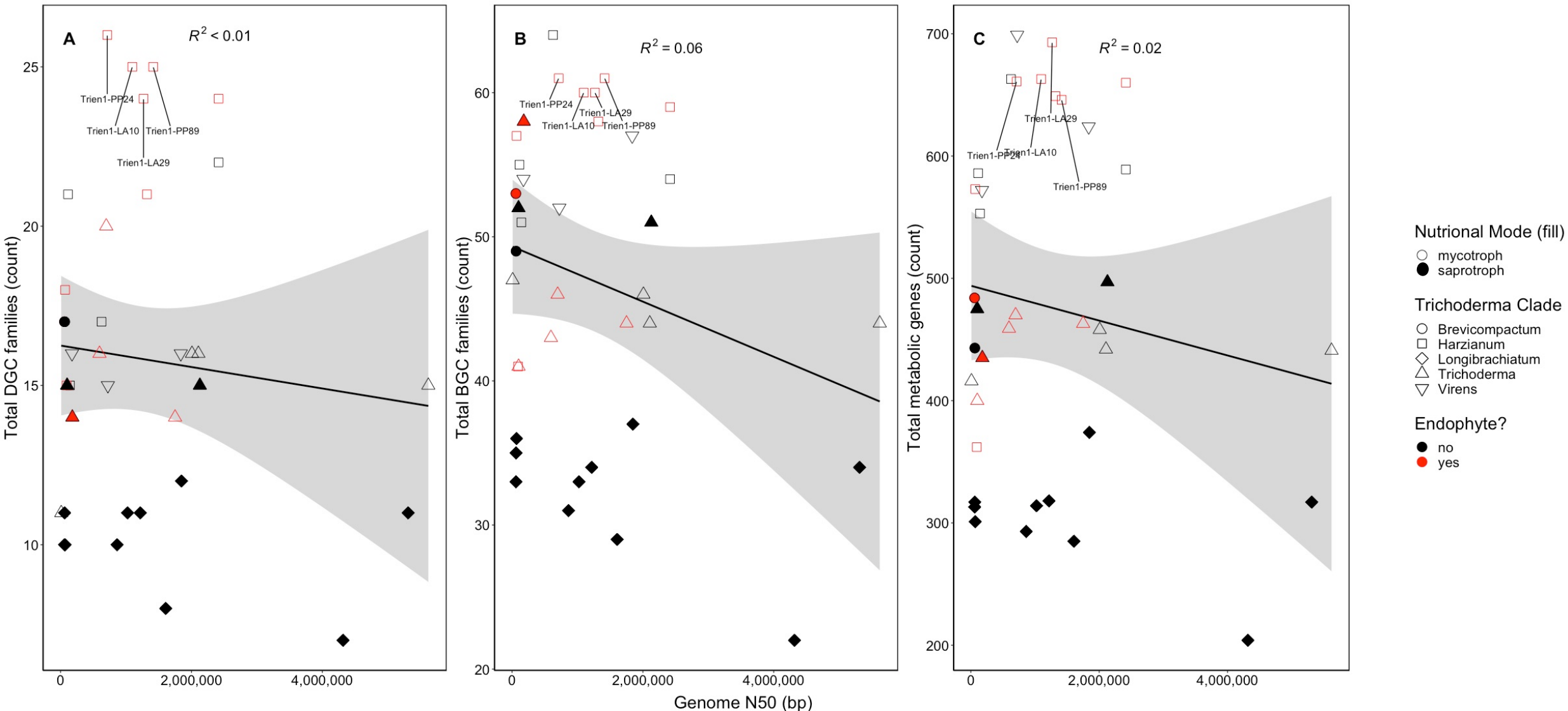

Supplement: S2 Fig — Genome quality is not correlated with the number of (A) DGC families (Pearson’s, p > 0.05) (B) BGC families (Pearson’s, p > 0.05), and (C) number of metabolic genes (Pearson’s, p > 0.05) identified in the Trichoderma genomes. Each point represents a Trichoderma isolate. Point fill indicates nutritional mode, point shape indicates the clade to which the isolate belongs, and color indicates whether the Trichoderma species is recorded as having an endophytic lifestyle. Gray shaded areas indicate the 95% confidence interval. (PDF) [file pone.0289280.s002.pdf]

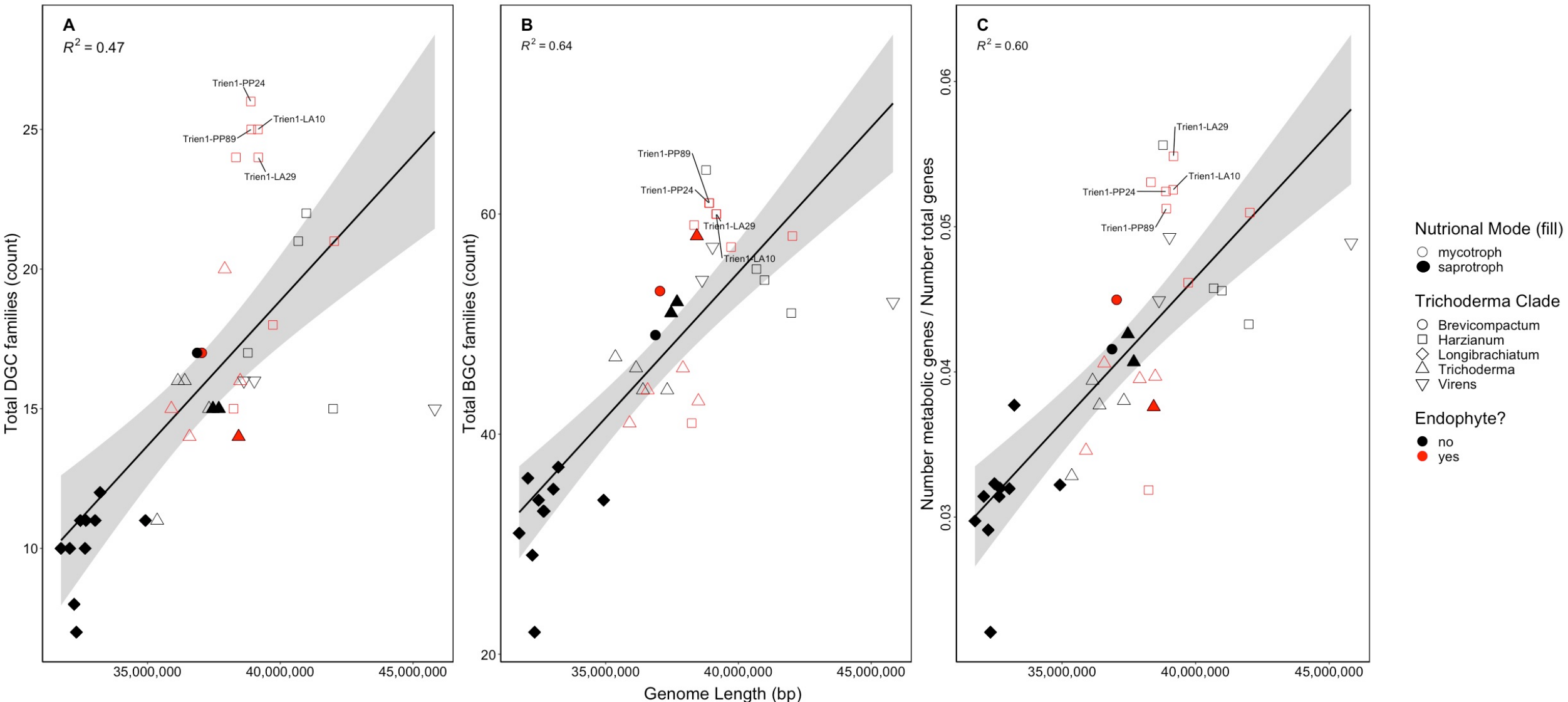

Supplement: S4 Fig — There is a significant correlation between genome length (bp) of the 38 Trichoderma genomes and the diversity of (A) DGC families (Pearson’s, p ≤ 0.05), (B) BGC families (Pearson’s, p ≤ 0.05), and (C) proportion of metabolic genes to total genes (Pearson’s, p ≤ 0.05). Points highlighted in red are isolates identified to have an endophytic lifestyle, points in black did not have an identified endophytic lifestyle, and points in blue were species with a potential endophytic lifestyle. Mycotrophic genomes are represented by empty points, saprotrophic genomes are represented by filled points. Gray shaded areas indicate the 95% confidence interval. (PDF) [file pone.0289280.s004.pdf]

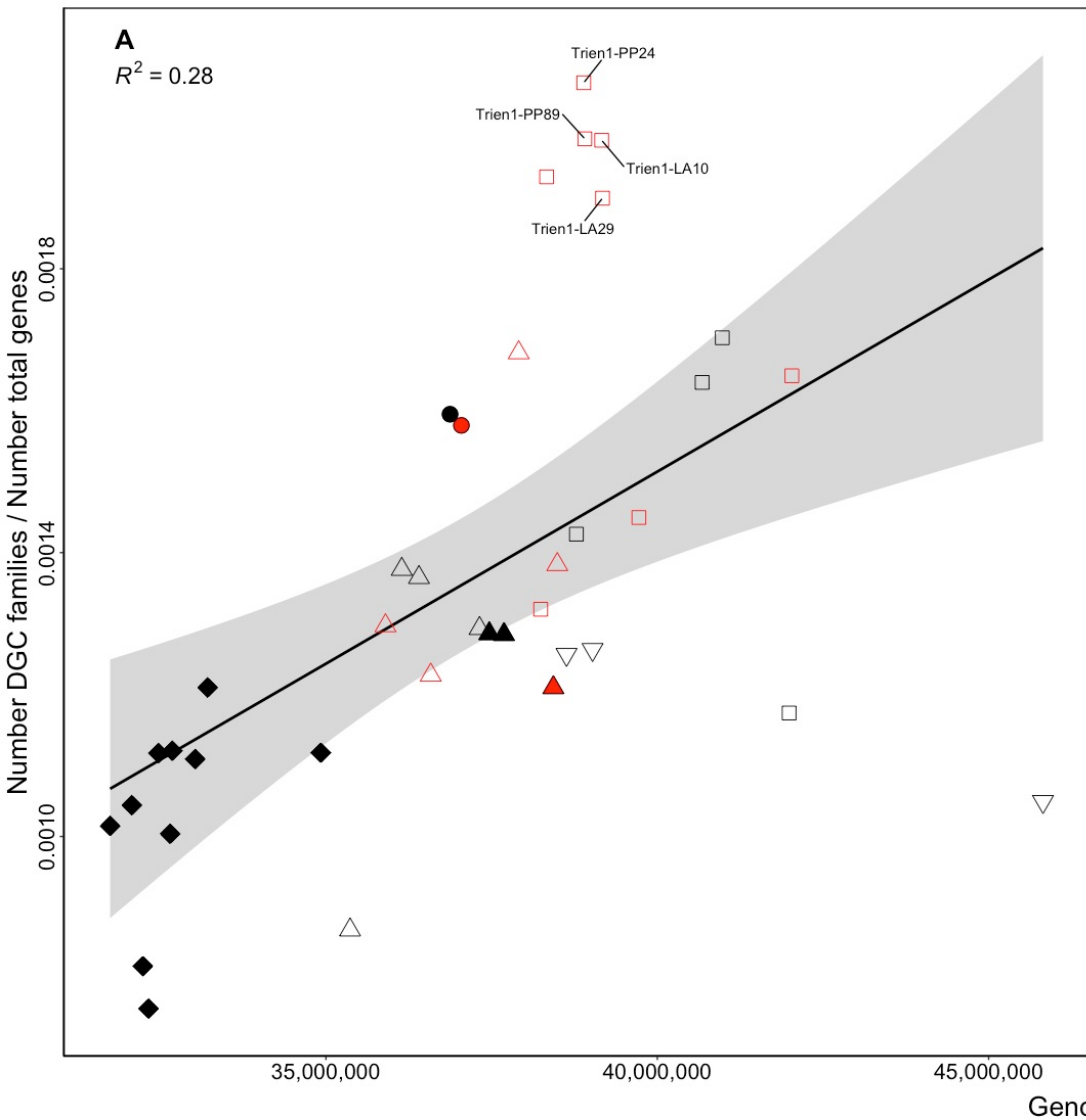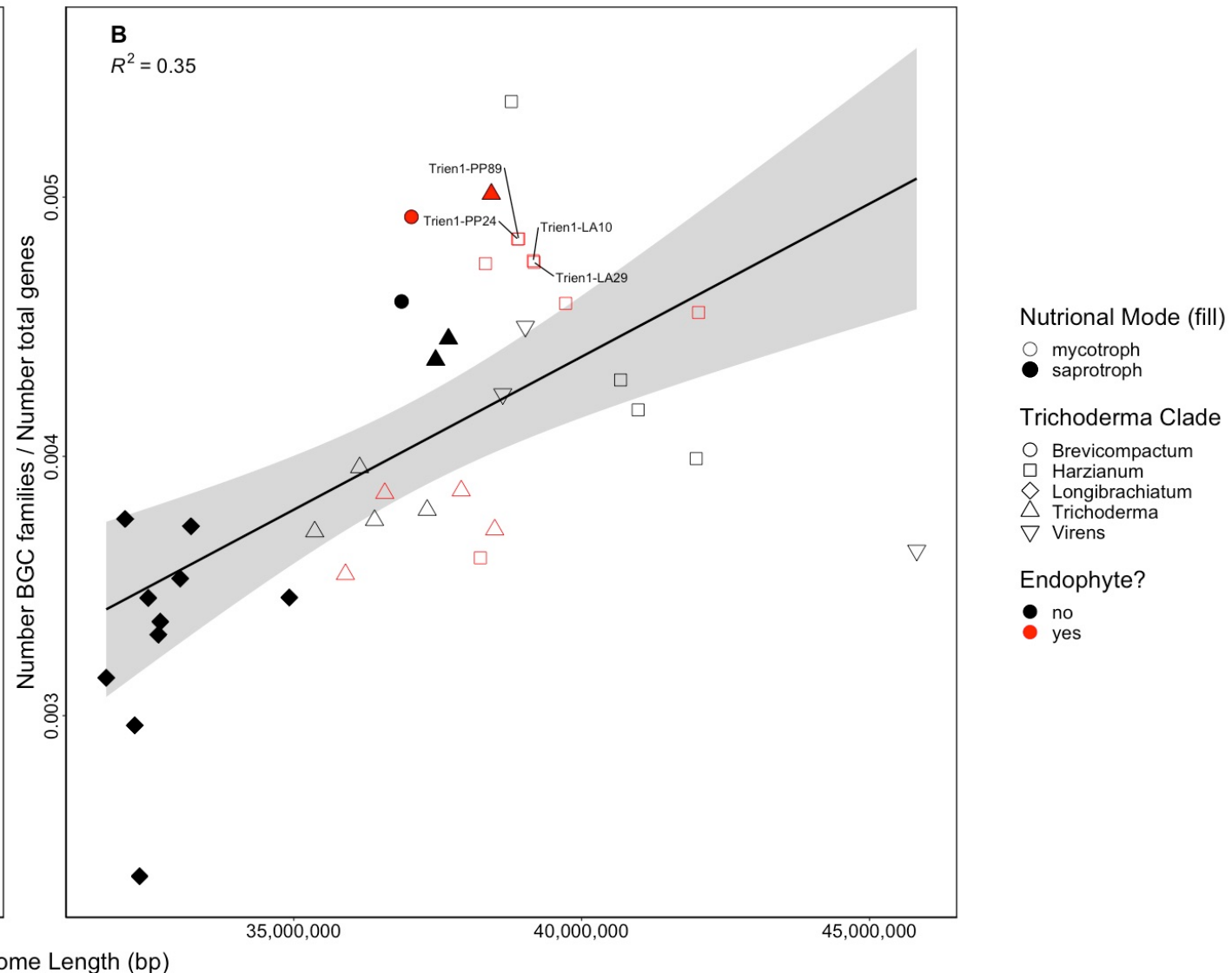

Supplement: S5 Fig — There is a significant correlation between genome length (bp) of the 38 Trichoderma genomes and the diversity of (A) proportion of biosynthetic genes to BGC clusters (Pearson’s, p ≤ 0.05) and (B) proportion of DGC families to total genes (Pearson’s, p ≤ 0.05). Points highlighted in red are isolates identified to have an endophytic lifestyle, points in black did not have an identified endophytic lifestyle, and points in blue were species with a potential endophytic lifestyle. Mycotrophic genomes are represented by empty points, saprotrophic genomes are represented by filled points. Gray shaded areas indicate the 95% confidence interval. (PDF) [file pone.0289280.s005.pdf]

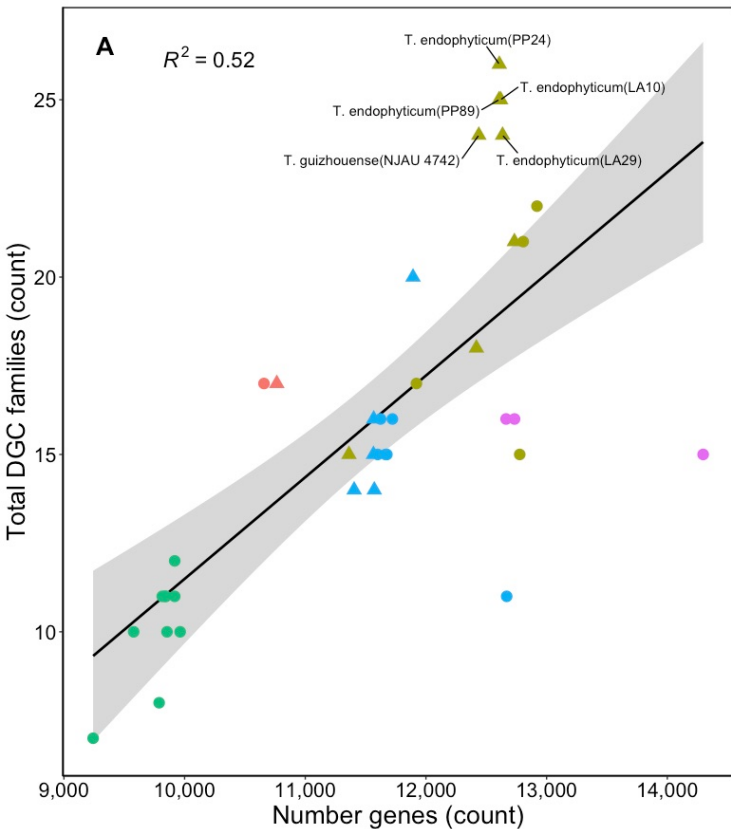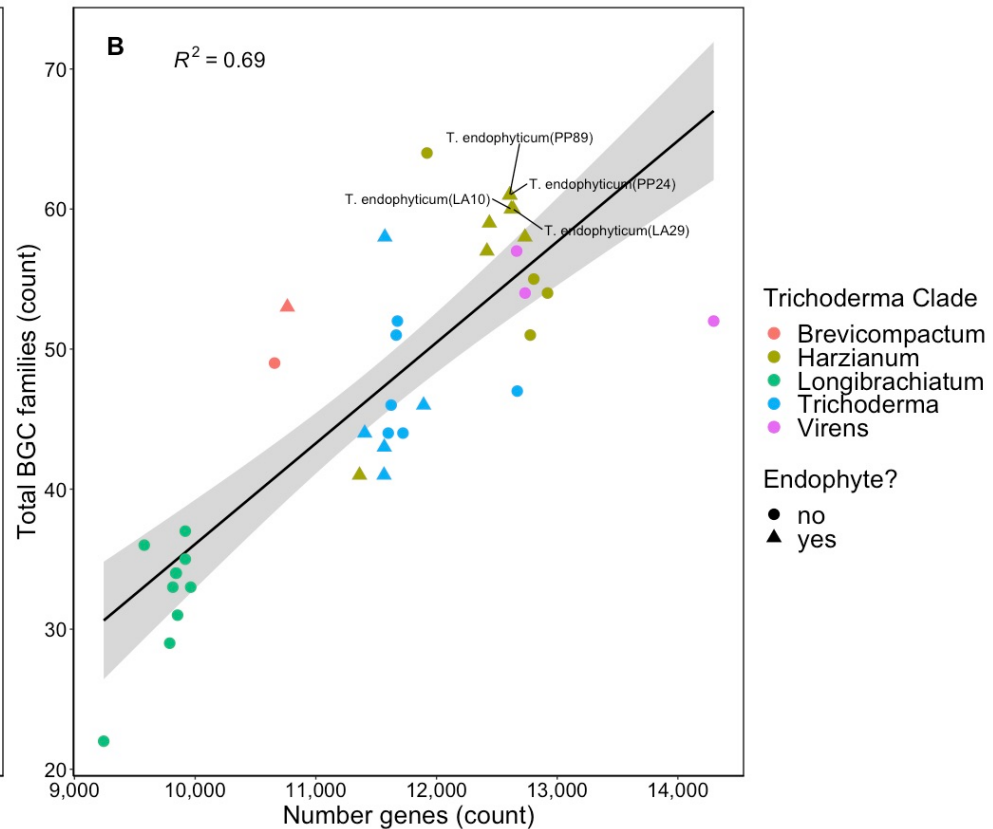

Supplement: S6 Fig — The overall number of genes identified in a Trichoderma genome is correlated with the number of (A) identified DGCs (Pearson’s, p < 0.005) and (B) BGCs (Pearson’s, p < 0.005). Gray shaded areas indicate the 95% confidence interval. (PDF) [file pone.0289280.s006.pdf]

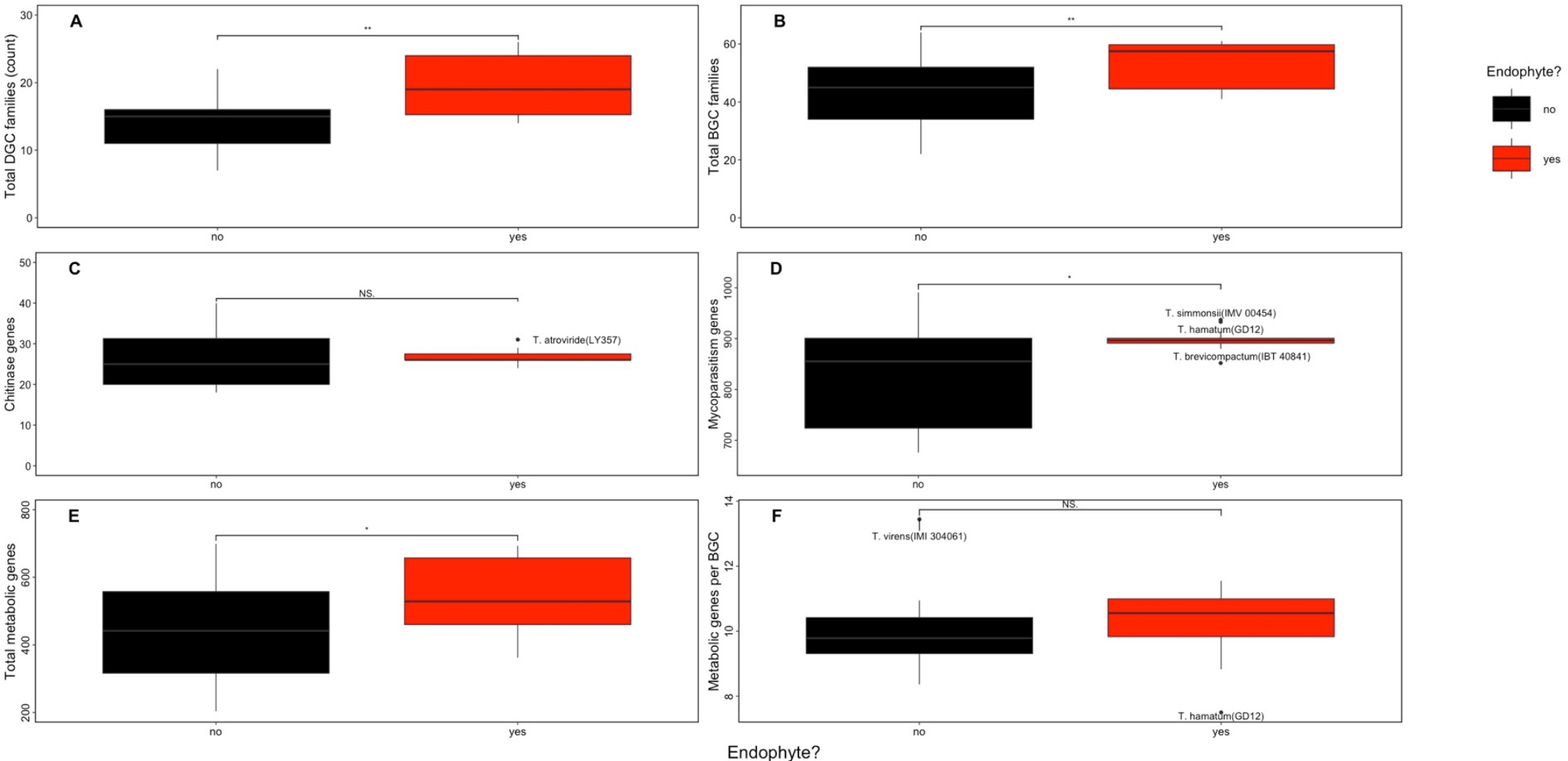

Supplement: S7 Fig — The average number metabolic gene clusters is significantly different between endophytic and non-endophytic Trichoderma genomes for both (A) DGCs (Wilcoxon, p ≤ 0.05) and (B) BGCs (two-sample t-test, p ≤ 0.05). There is no significant differences between the number of (C) chitinase genes for endophytic and non-endophytic Trichoderma genomes (Wilcoxon, p > 0.05). The number of mycoparasitism genes is significantly higher in (D) endophyte genomes (Wilcoxon, p ≤ 0.05). (E) The number of metabolic genes per genome were higher in endophytes than in non-endophyte genomes (two-sample t-test, p ≤ 0.05). (F) The number of metabolic genes per BGC was not significantly different between endophyte and non-endophyte genomes (two-sample t-test, p > 0.05). (PDF) [file pone.0289280.s007.pdf]

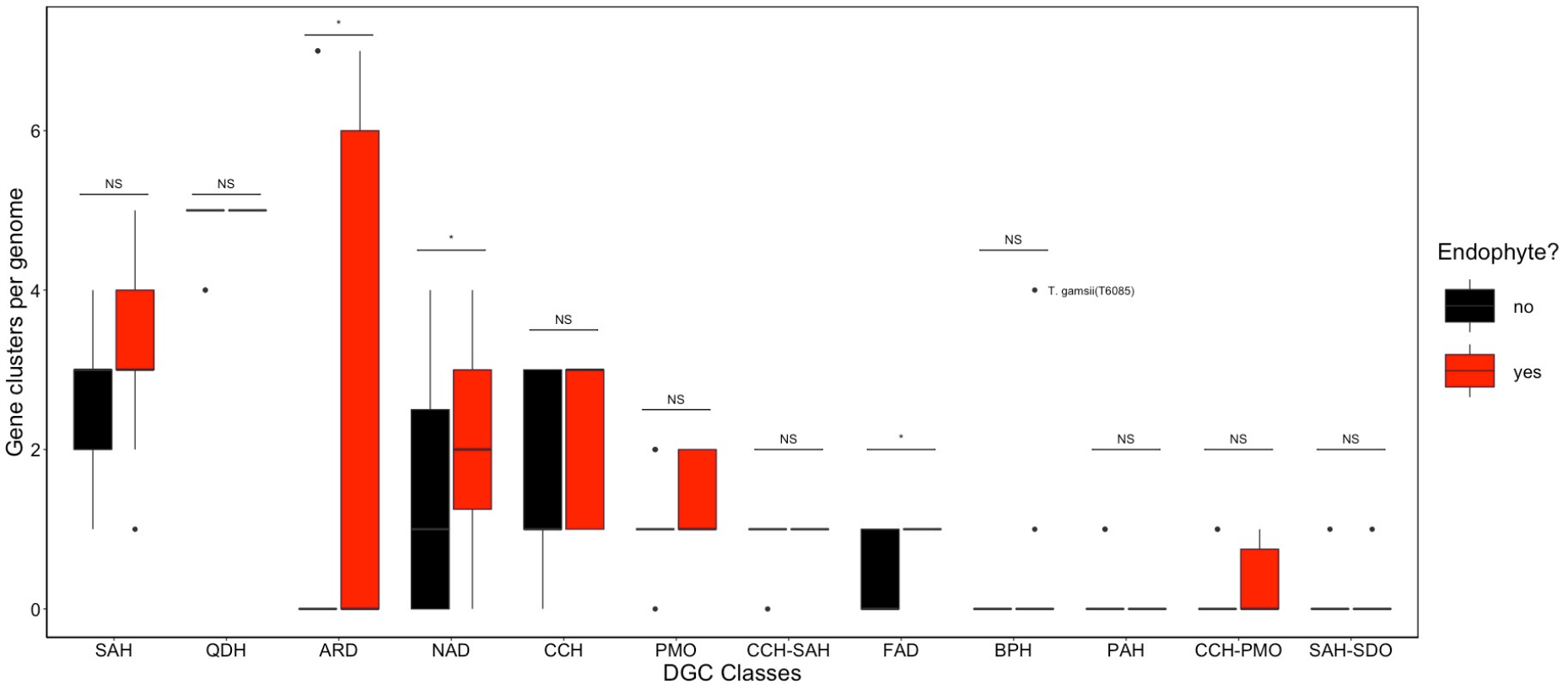

Supplement: S8 Fig — Differences between endophytic and non-endophytic DGC class counts were calculated using Wilcoxon rank sum test corrected with Holm-Bonferroni method. Endophytic genomes have significantly greater ARD, NAD, and FAD (each p ≤ 0.05), significance differences between groups are indicated with an asterisk. No significant difference between endophyte and non-endophyte DGC count is indicated with “NS” (p > 0.05). (PDF) [file pone.0289280.s008.pdf]

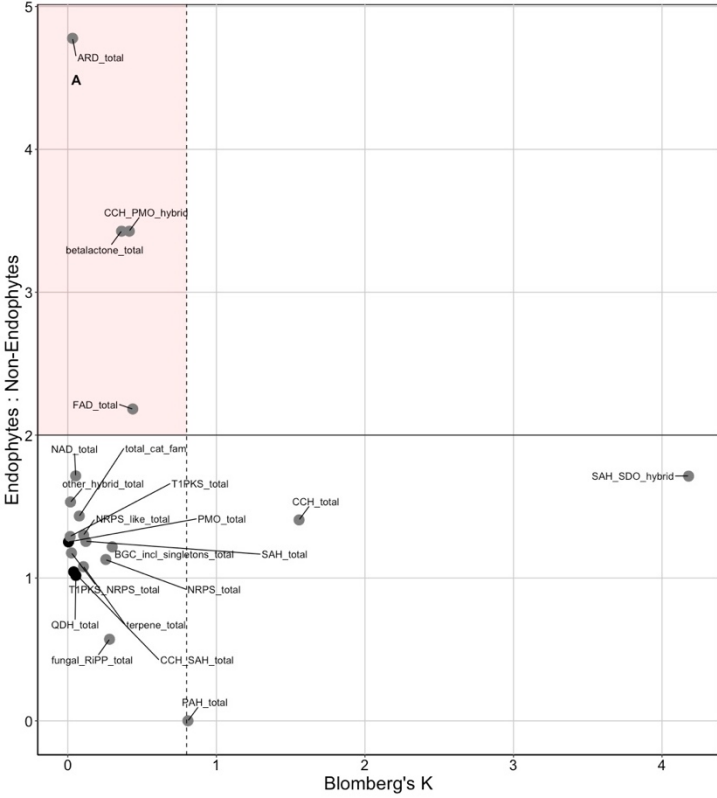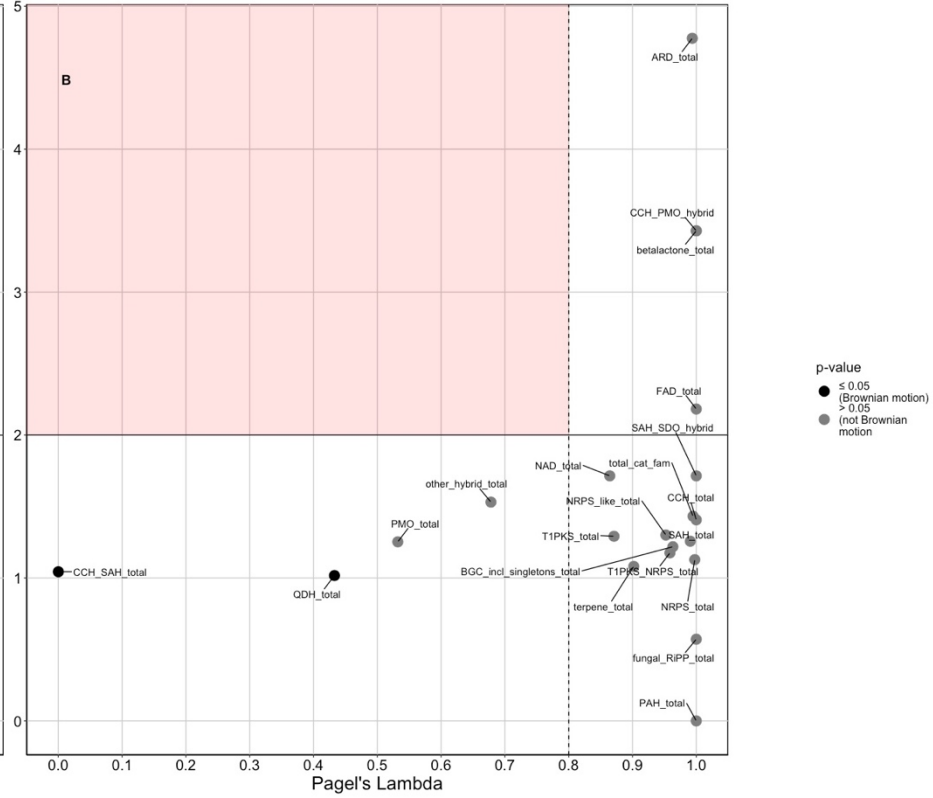

Supplement: S9 Fig — Most evaluated DGC and BGC class distributions were determined to be consistent with Brownian motion evolution as determined by their (A) Blomberg’s K value and significance (p-value ≤ 0.05) and (B) Pagel’s Lambda value and significance (p ≤ 0.05). Black points in the shaded area of the graph would indicate DGC classes which exhibit overdispersal in the Trichoderma phylogeny as well as overrepresentation in endophytic genomes. (PDF) [file pone.0289280.s009.pdf]

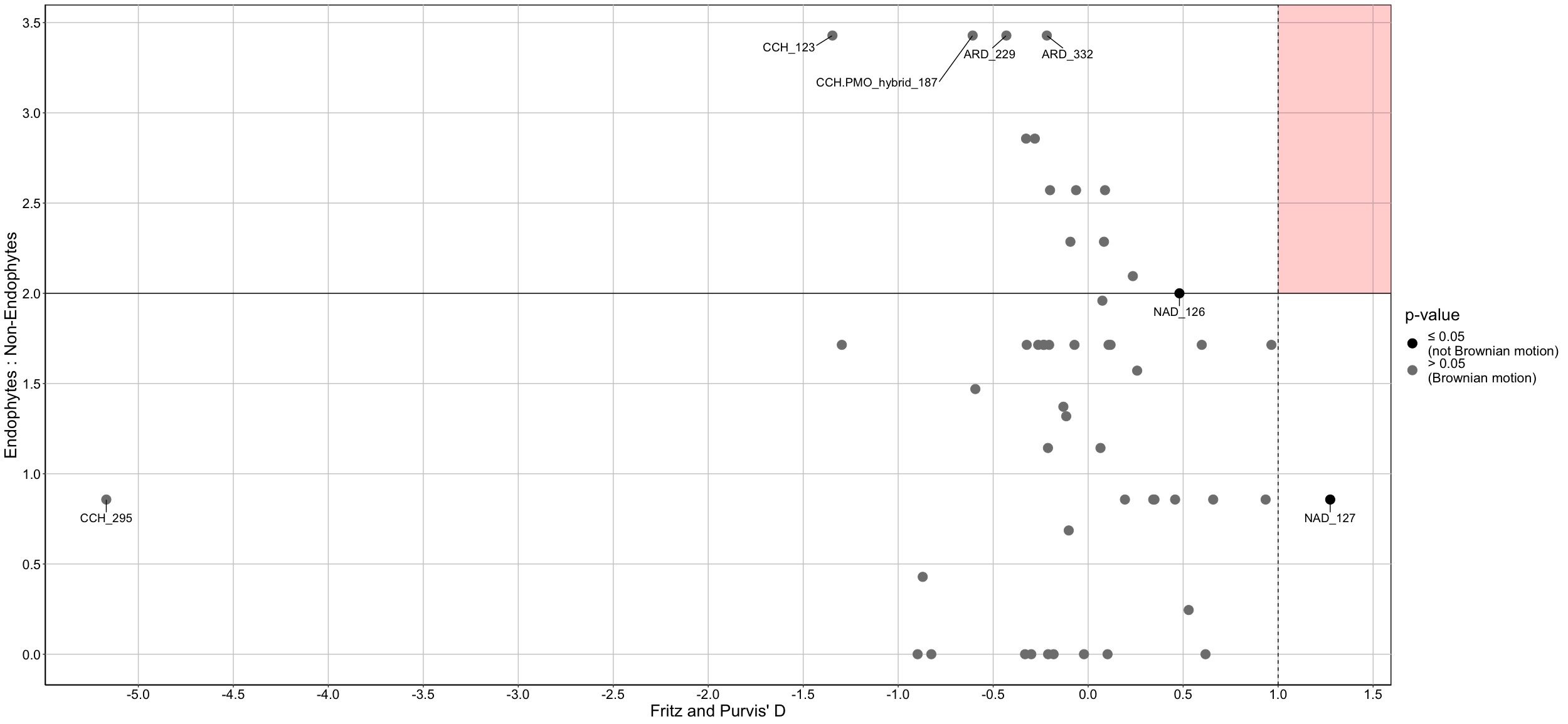

Supplement: S10 Fig — Most DGC families did not have a significant result when analyzing each DGC’s Fritz and Purvis’ D-statistic (p > 0.05), indicating that the distribution most DGC families followed Brownian motion evolution. The DGC families most overrepresented in endophyte genomes (E:NE > 3), families with a significant D-statistic (p ≤ 0.05), families with an very low D-statistic (D-estimate < -2) are labeled. Black points in the shaded area of the graph would indicate DGC families which exhibit overdispersal in the Trichoderma phylogeny as well as overrepresentation in endophytic genomes. (JPG) [file pone.0289280.s010.jpg]

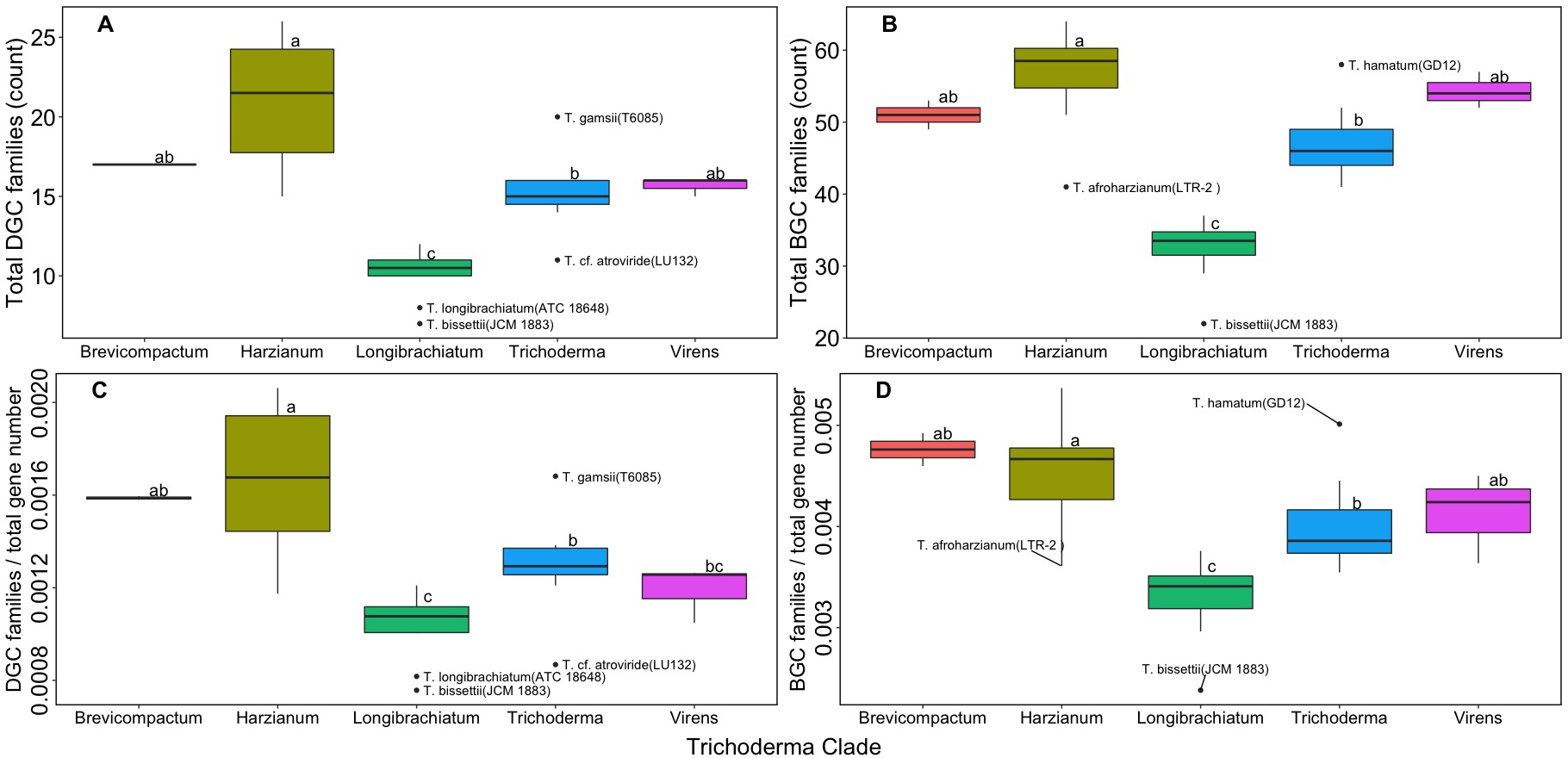

Supplement: S11 Fig — There are significant differences between the Trichoderma clades for total count of (A) DGCs per genome (p ≤ 0.05) and (B) BGCs per genome (p ≤ 0.05). There are also significant differences between clades for the (C) proportion of DGCs to total gene number in each genome (p ≤ 0.05) and (D) proportion of BGCs to total gene number in each genome (p ≤ 0.05). Significant differences were determined using Kruskal-Wallis test, post hoc differences were determined with Dunn’s test with Bonferroni correction. (JPG) [file pone.0289280.s011.jpg]

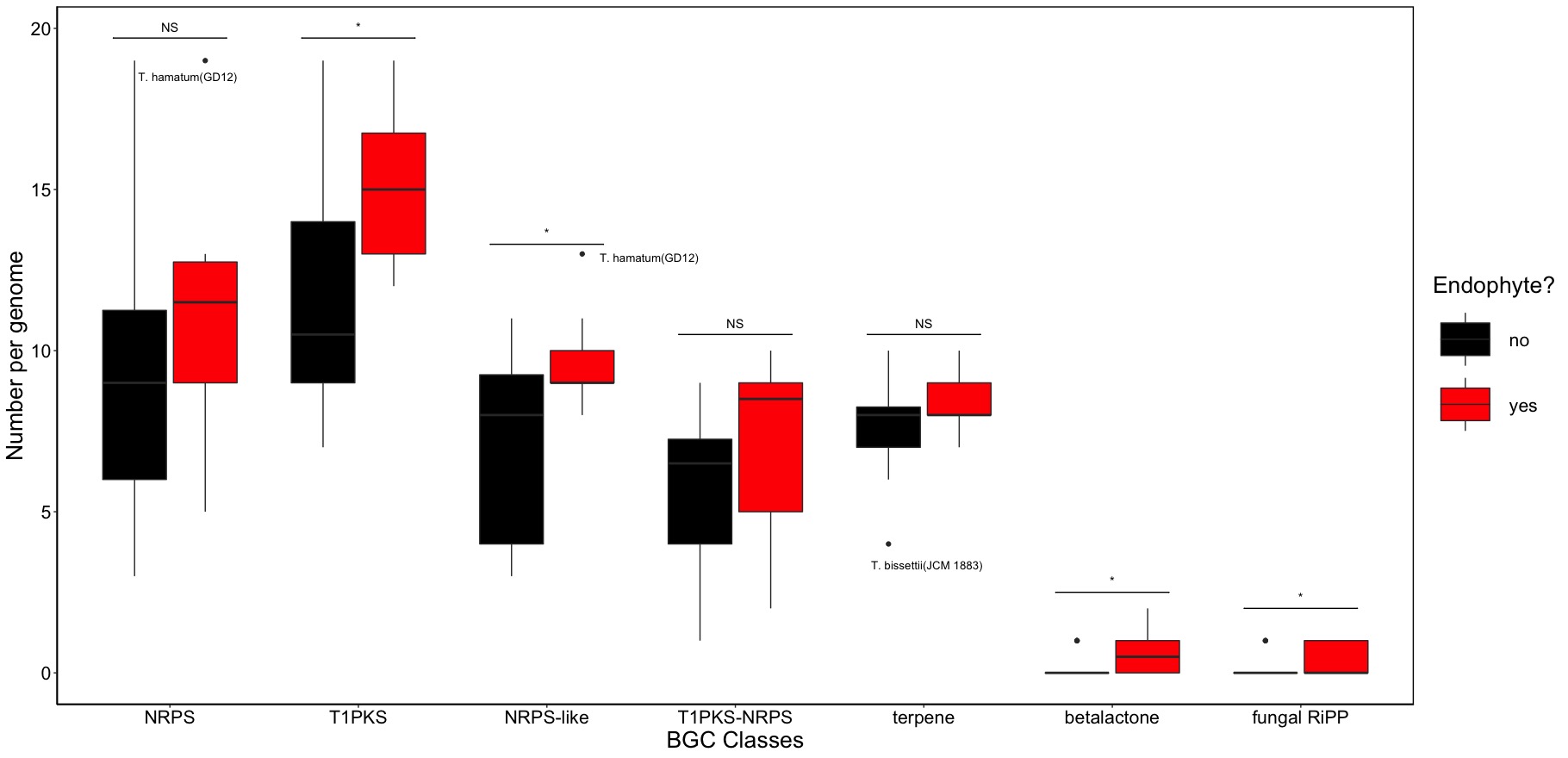

Supplement: S13 Fig — Significant differences were determined by the Wilcoxon rank sum test corrected with Holm-Bonferroni method. No significant differences between groups are indicated by “NS”. Outliers within groups are labeled. (JPG) [file pone.0289280.s013.jpg]

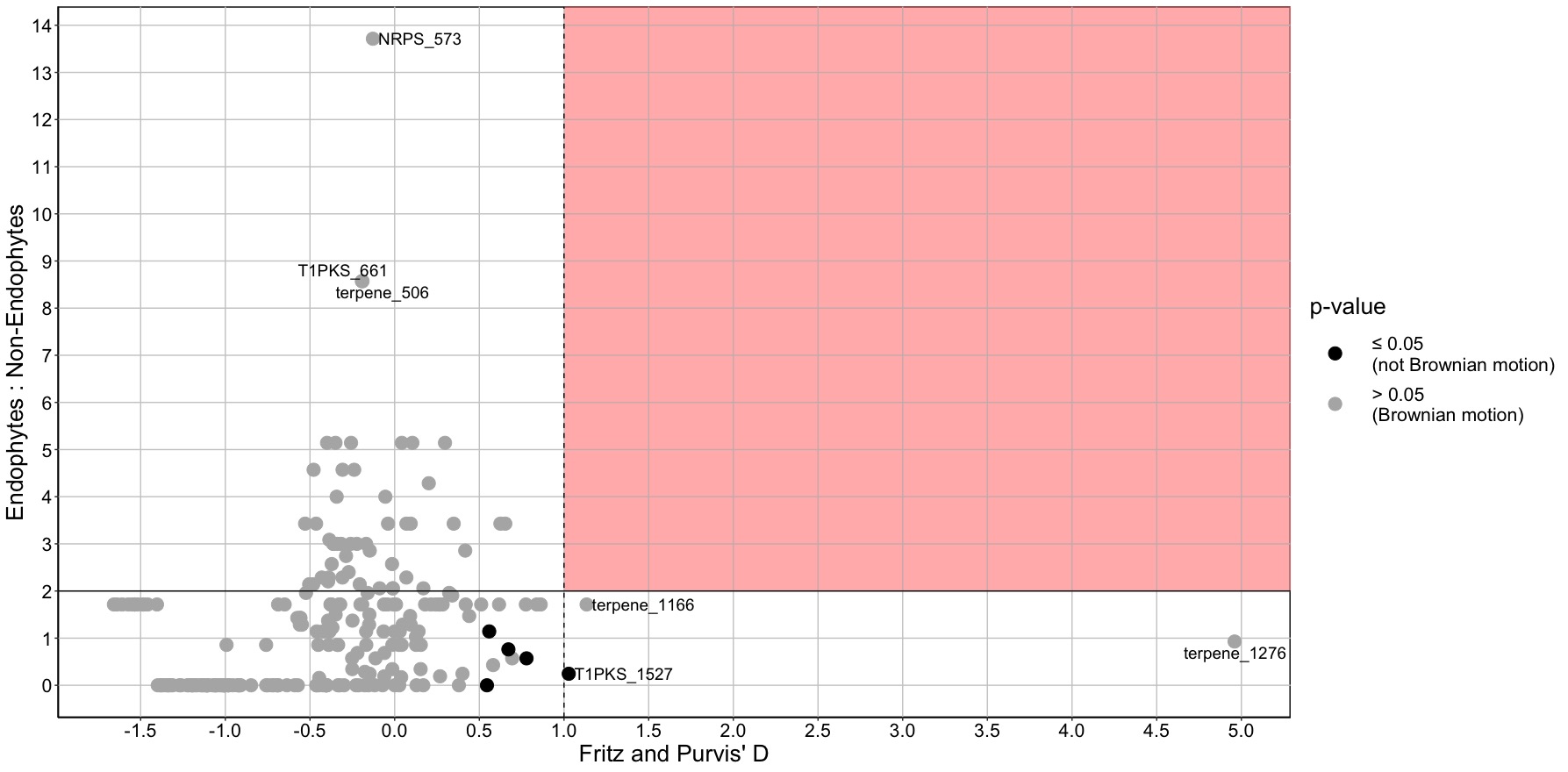

Supplement: S14 Fig — The majority of evaluated BGC families did not have a significant result when analyzing each’s Fritz and Purvis’ D-statistic (p > 0.05), which indicated Brownian motion evolution. The BGC families that did not exhibit Brownian motion model evolution were not found enriched in endophyte genomes (E:NE < 2). The BGC families most overrepresented in endophyte (E:NE value > 6) and DGCs overdispersed in the Trichoderma phylogeny (D-statistic > 1) are labeled. Black points in the shaded area of the graph would indicate BGC families which exhibit overdispersal in the Trichoderma phylogeny as well as overrepresentation in endophytic genomes. (JPG) [file pone.0289280.s014.jpg]

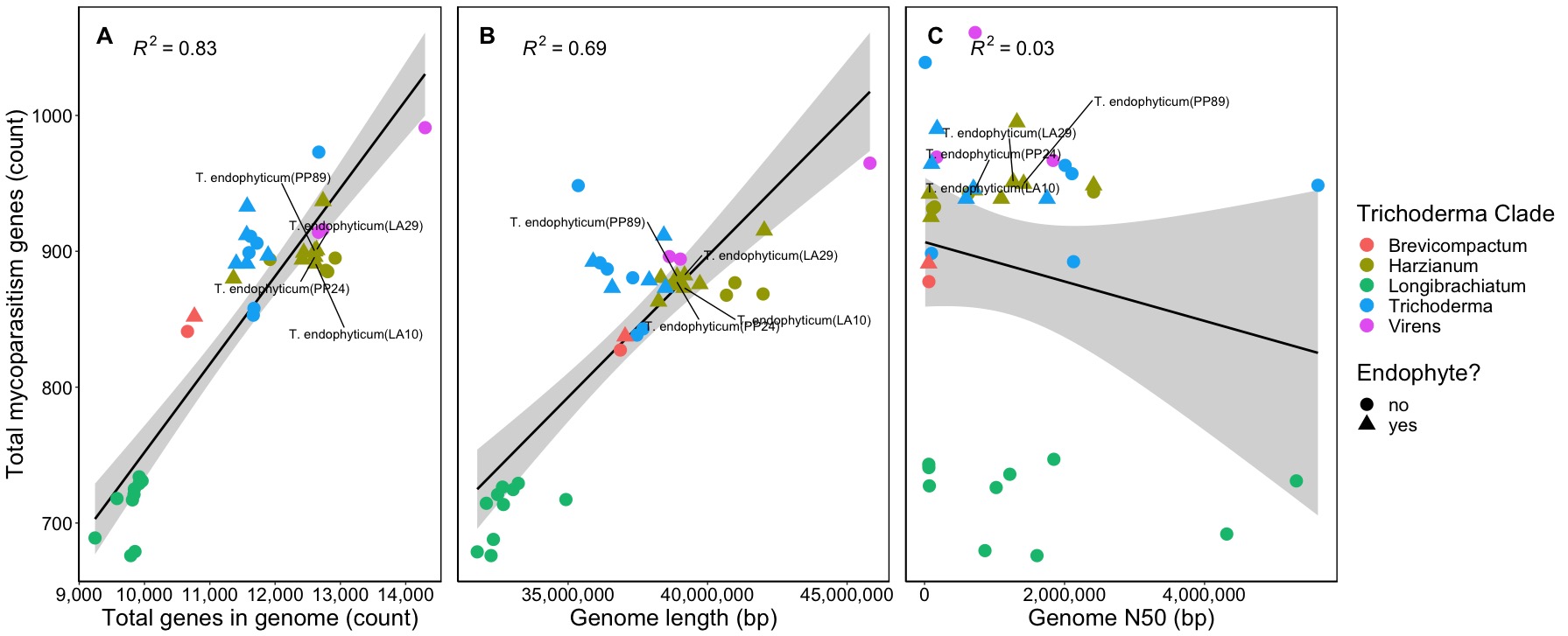

Supplement: S15 Fig — The total number of mycoparasitism genes per genome is positively correlated with (A) total genes in a Trichoderma genome (Pearson’s, p ≤ 0.05) and (B) genome length (Pearson’s, p ≤ 0.05). (C) There is no relation to genome quality (N50) and number of identified mycoparasitism-related genes per genome (Pearson’s, p > 0.05). Gray shaded areas indicate the 95% confidence interval. (JPG) [file pone.0289280.s015.jpg]

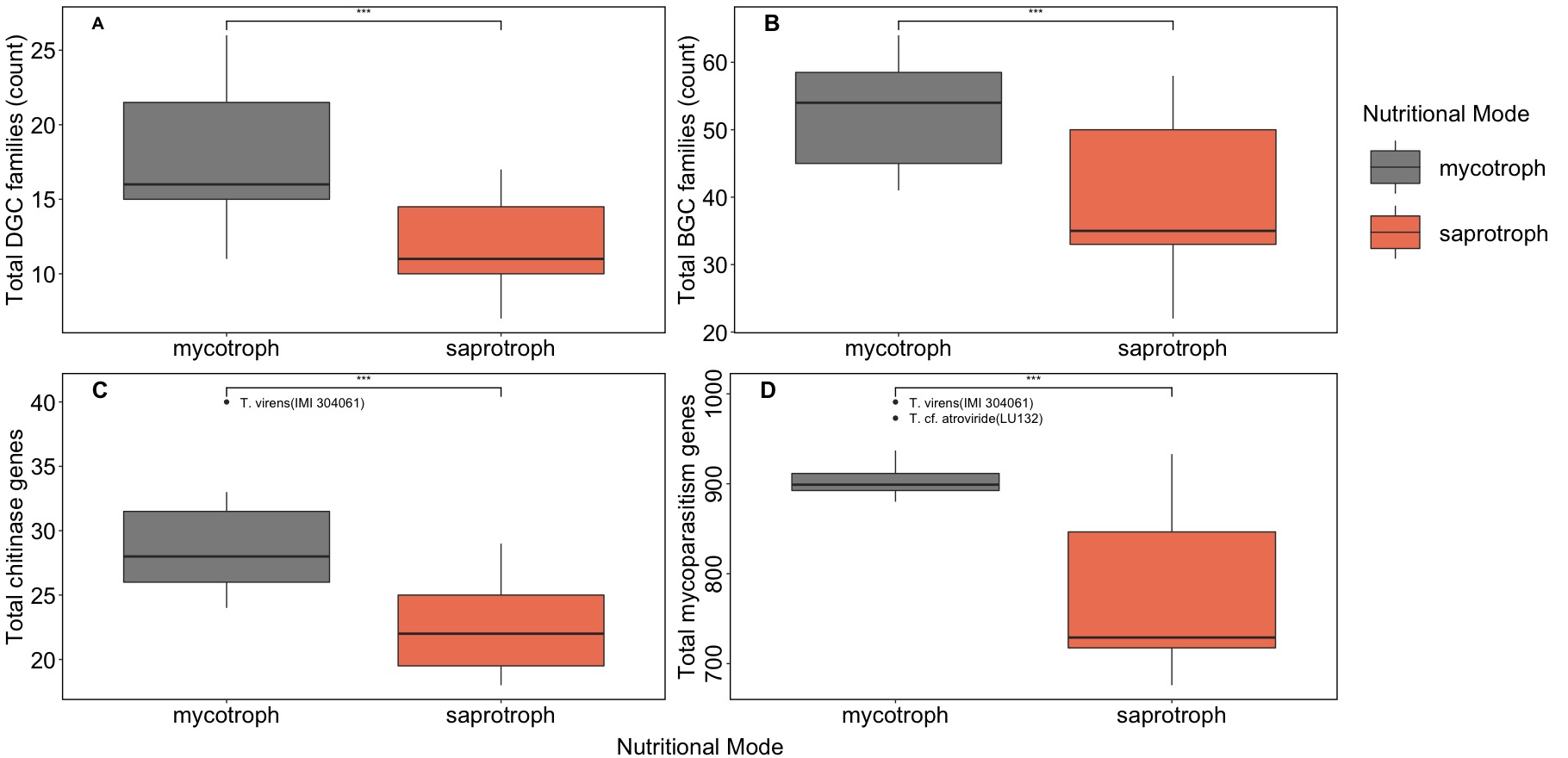

Supplement: S16 Fig — The average number of metabolic gene clusters is significantly different between mycotrophic and saprotrophic Trichoderma genomes for both A) DGCs (2-sample t-test, p ≤ 0.0005) and B) BGCs (2-sample t-test, p ≤ 0.0005). The total number of C) chitinase genes is higher in mycotrophic genomes (2-sample t-test, p ≤ 0.0005), as are the D) total number of mycoparasitism genes (2-sample t-test, p ≤ 0.0005). Outliers are labeled and significant differences are indicated with asterisks. (JPG) [file pone.0289280.s016.jpg]

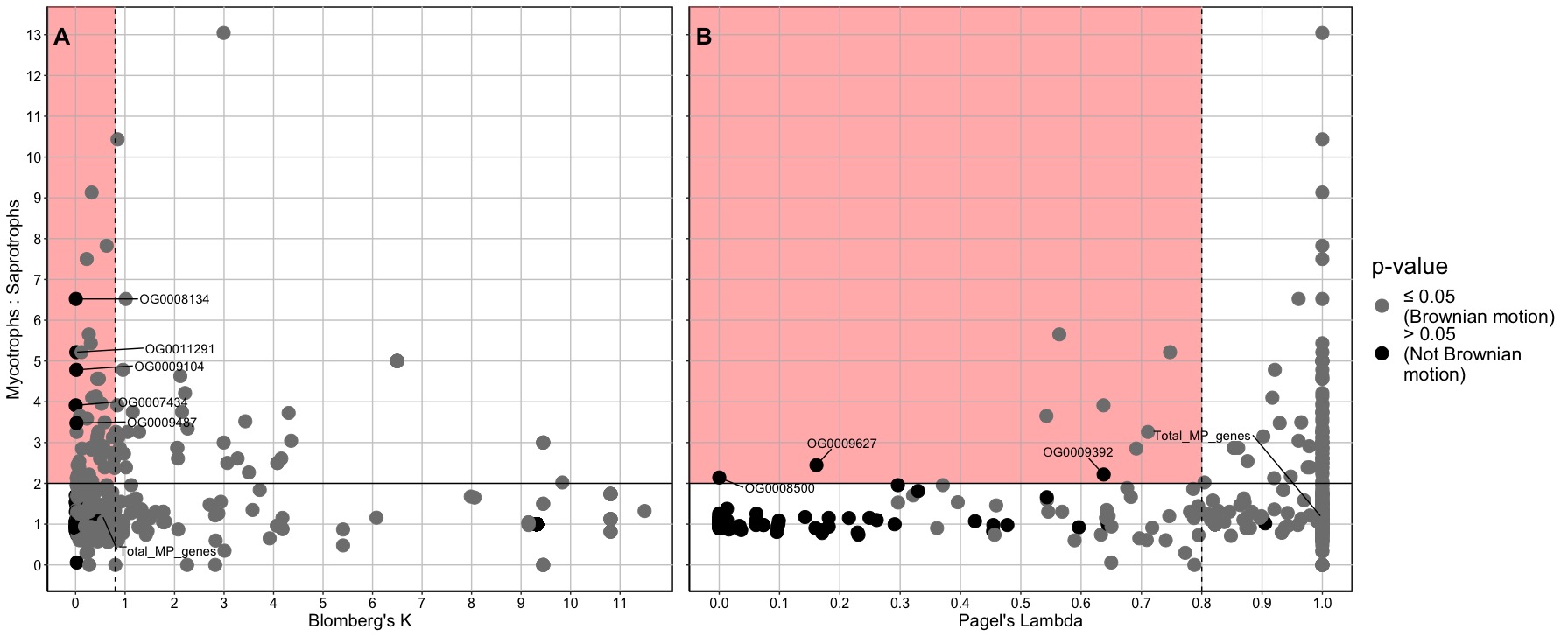

Supplement: S17 Fig — (A) The mycotroph to saprotroph gene ratio (M:S) and Blomberg’s K value suggests that five mycoparasitism genes are overdispersed in Trichoderma (K < 0.8, p > 0.05) and enriched in genomes with a mycotrophic nutritional mode (M:S > 2). (B) The mycotroph to saprotroph gene ratio (M:S) and Blomberg’s K value suggests that three mycoparasitism genes are overdispersed in Trichoderma (Lambda < 0.8, p > 0.05) and enriched in genomes with a mycotrophic nutritional mode (M:S > 2). Black points in the shaded area of the graph would indicate mycoparasite genes which exhibit overdispersal in the Trichoderma phylogeny as well as overrepresentation in mycotrophic genomes. (JPG) [file pone.0289280.s017.jpg]

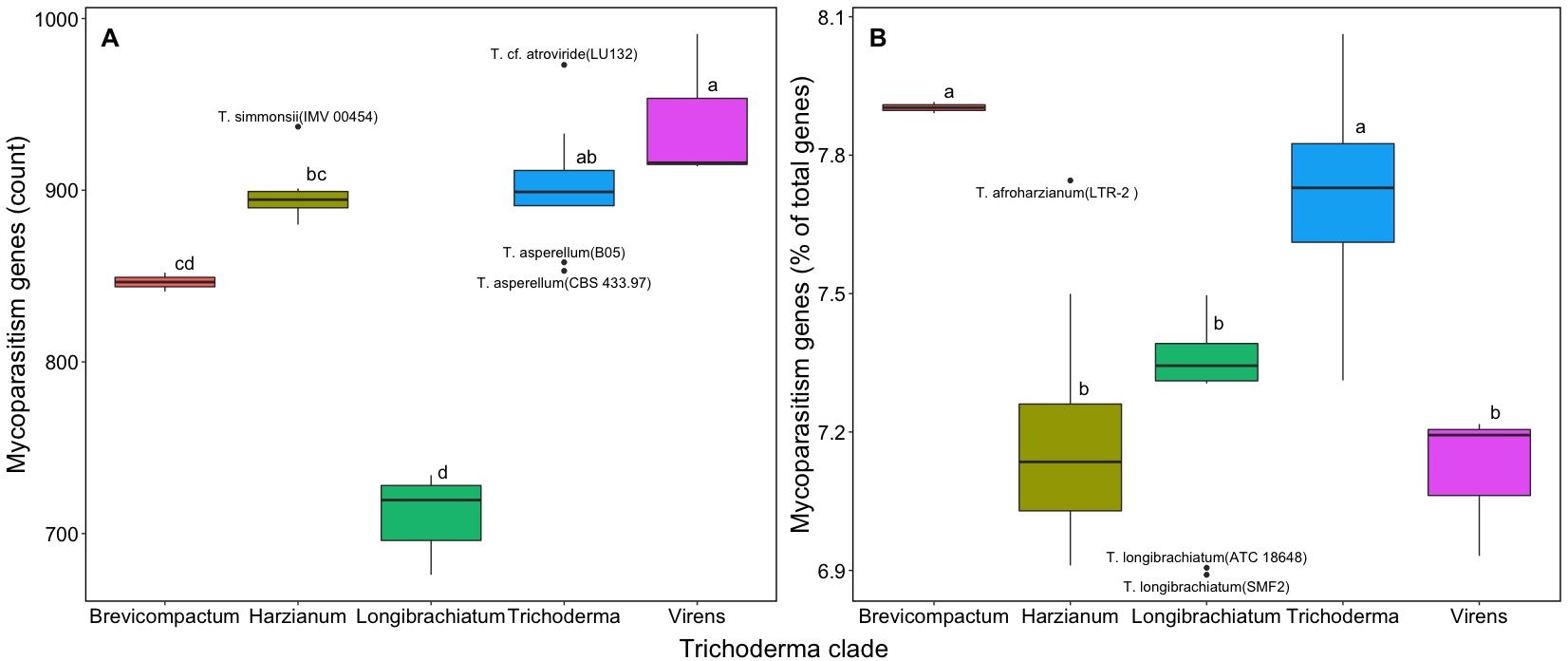

Supplement: S18 Fig — (A) Longibrachiatum contains the fewest amount of mycoparasitism-related genes while Virens and section Trichoderma contain the highest amount (Kruskal-Wallis, correction with Bonferroni, p ≤ 0.05). (B) Harzianum, Longibrachiatum, and Virens contain the lowest percent of mycoparasitism genes to the total number of genes per genome, and Brevicompactum and section Trichoderma contains the highest (ANOVA, correction with Bonferroni, p ≤ 0.05). (JPG) [file pone.0289280.s018.jpg]

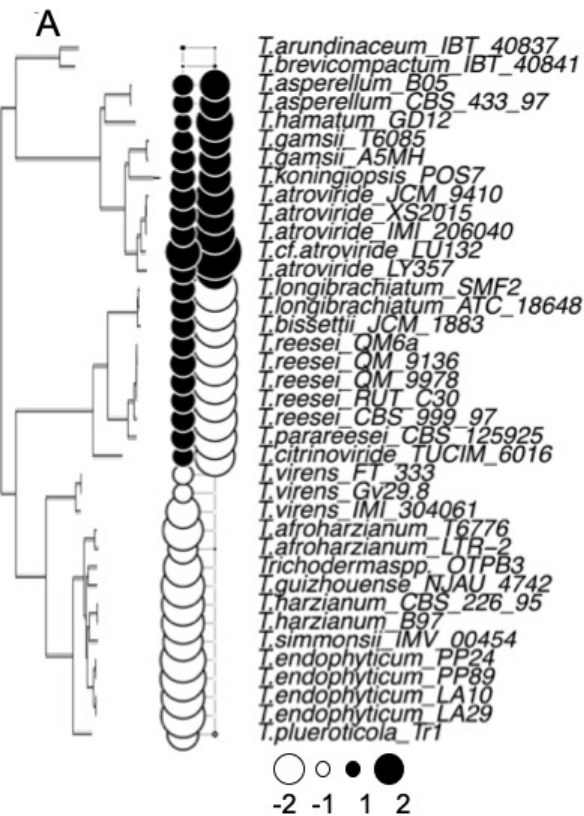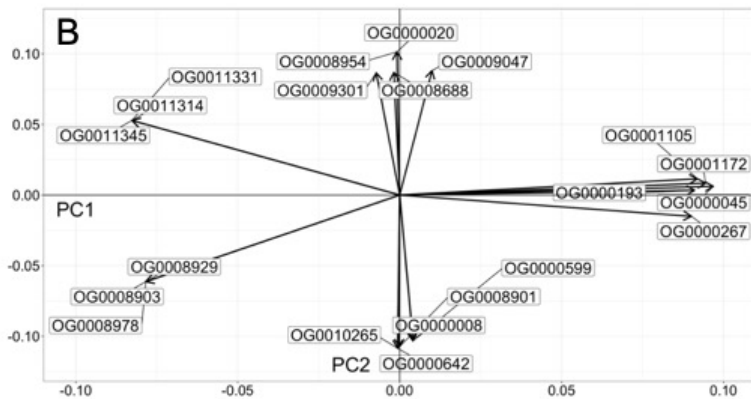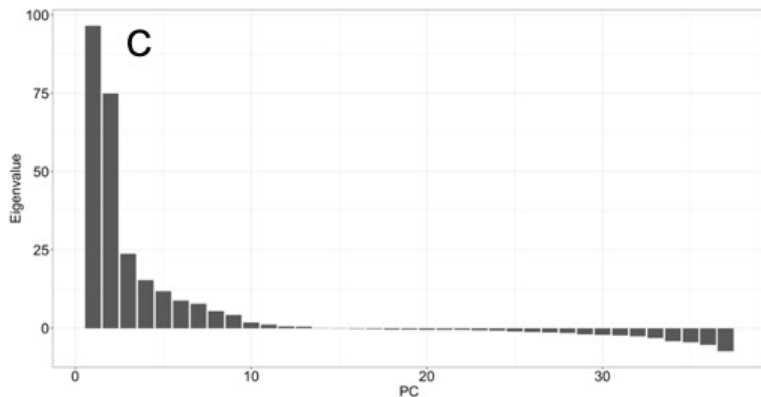

Supplement: S19 Fig — (A) Phylogenetic tree and relative signal of phylogenetically-corrected global principal component 1 (PC1) and global principal component 2 (PC2) for each Trichoderma genome. (B) Top 10 mycoparasitism genes contributing to PC1 signal and top 11 mycoparasitism genes contributing to PC2 signal. (C) Eigenvalues for the phylogenic correction PCA (pPCA) global principal components (PCs). (PDF) [file pone.0289280.s019.pdf]

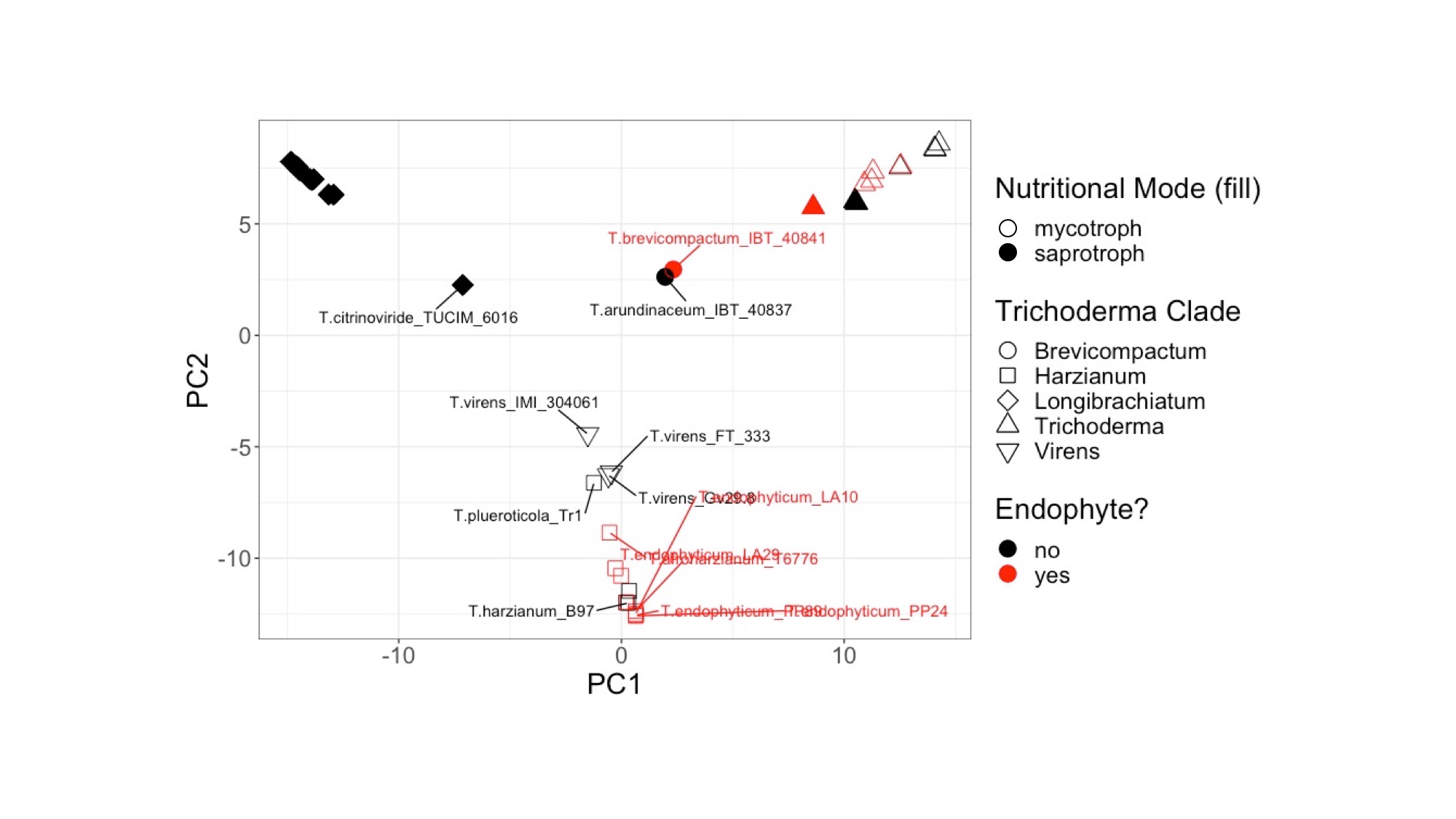

Supplement: S20 Fig — Principal components analysis (PCA) of the distribution of the 746 mycoparasitism-related genes; the inherent phylogenetic bias within the Trichoderma dataset was corrected with phylogenetic principal components analysis (pPCA). Plotting of the global principal components values for each genome shows that Trichoderma genomes are grouping based on Trichoderma clade rather than nutritional mode or lifestyle. (JPG) [file pone.0289280.s020.jpg]

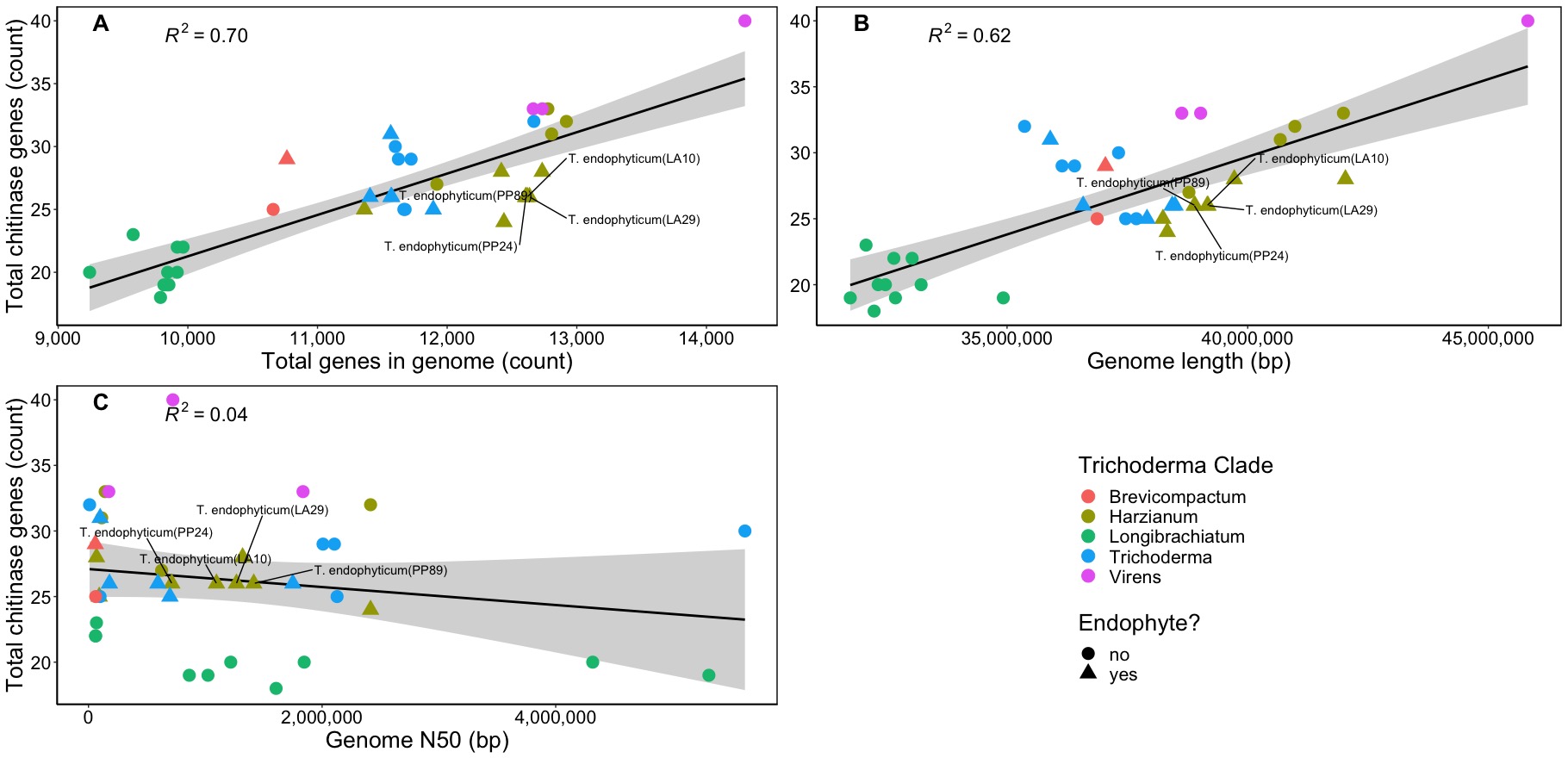

Supplement: S21 Fig — The overall number of genes identified in a Trichoderma genome is correlated with the number of A) identified chitinase genes (Pearson’s, p ≤ 0.05) and B) genome length (Pearson’s, p ≤ 0.05), but not with C) genome quality (Pearson’s, p ≥ 0.05). Gray shaded areas indicate the 95% confidence interval. (JPG) [file pone.0289280.s021.jpg]

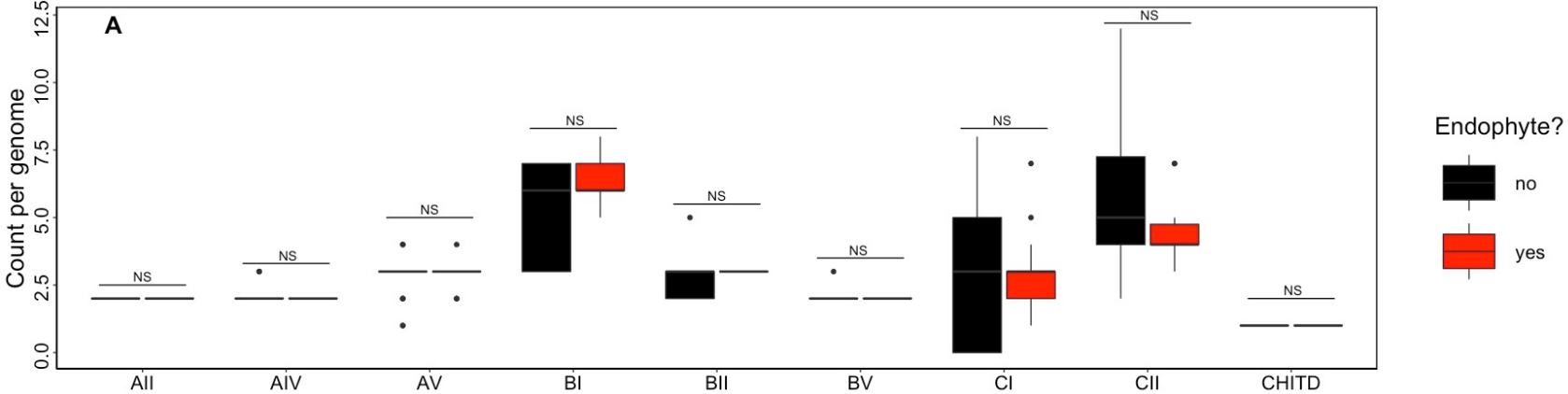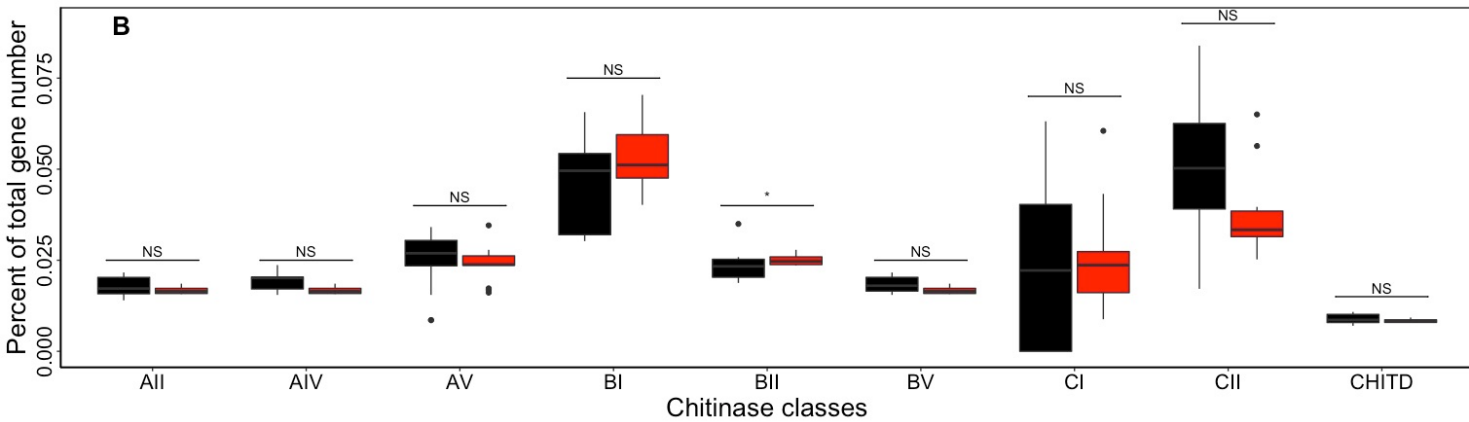

Supplement: S22 Fig — (A) Endophyte genomes do not significantly differ in any chitinase class overall count when compared with non-endophyte genomes (Wilcoxon, p > 0.05) (B) (A) Endophyte genomes only have a significantly higher proportion of chitinase class BII genes to total genes per genome as compared to non-endophyte genomes (Wilcoxon, p ≤ 0.05). (PDF) [file pone.0289280.s022.pdf]

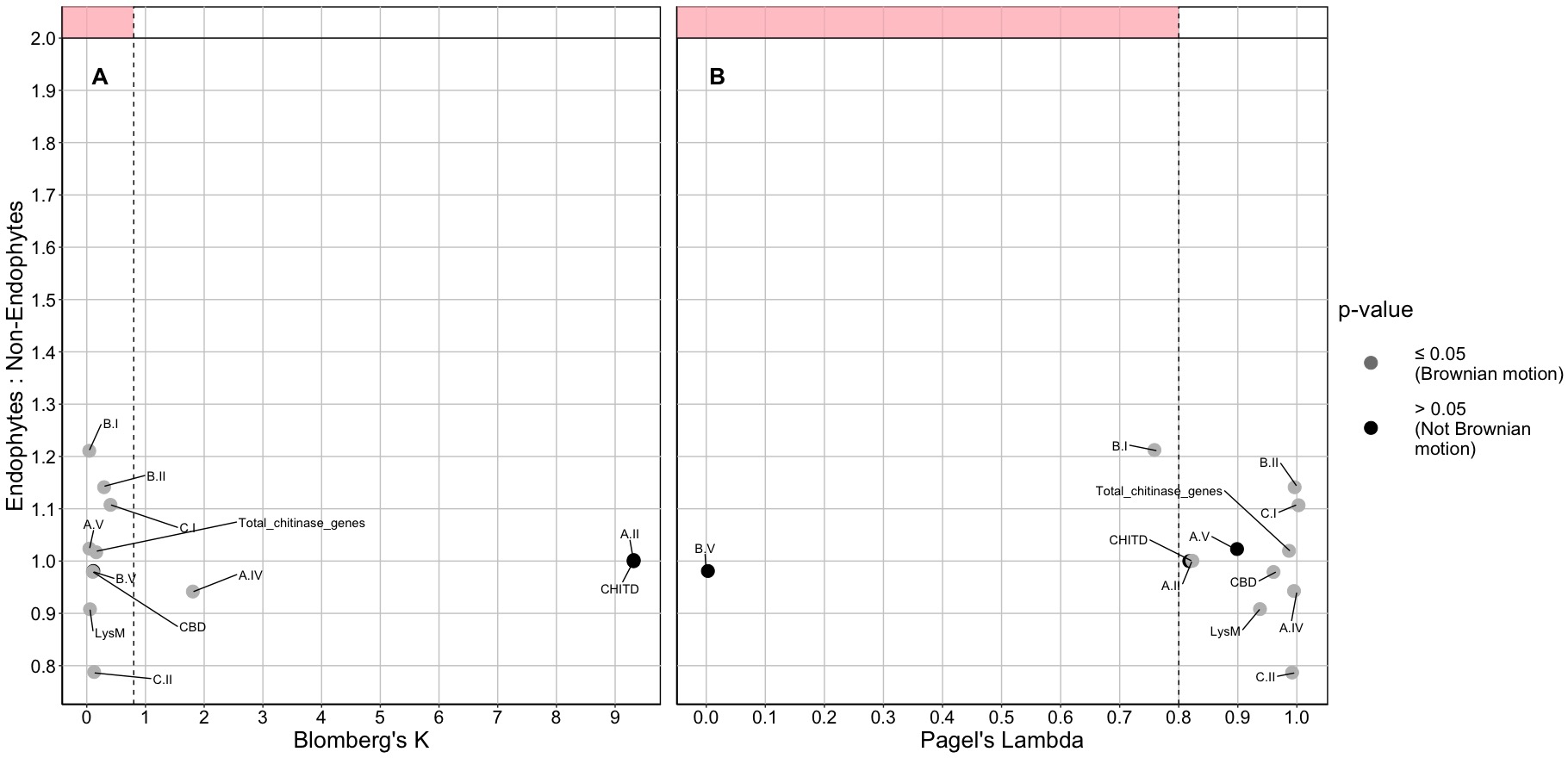

Supplement: S23 Fig — Most evaluated chitinase class distributions were determined to be consistent with Brownian motion evolution as determined by their (A) Blomberg’s K value and significance (p-value ≤ 0.05) and (B) Pagel’s Lambda value and significance (p ≤ 0.05). Black points in the shaded area of the graph would indicate chitinase gene classes which exhibit overdispersal in the Trichoderma phylogeny as well as overrepresentation in endophytic genomes. (JPG) [file pone.0289280.s023.jpg]

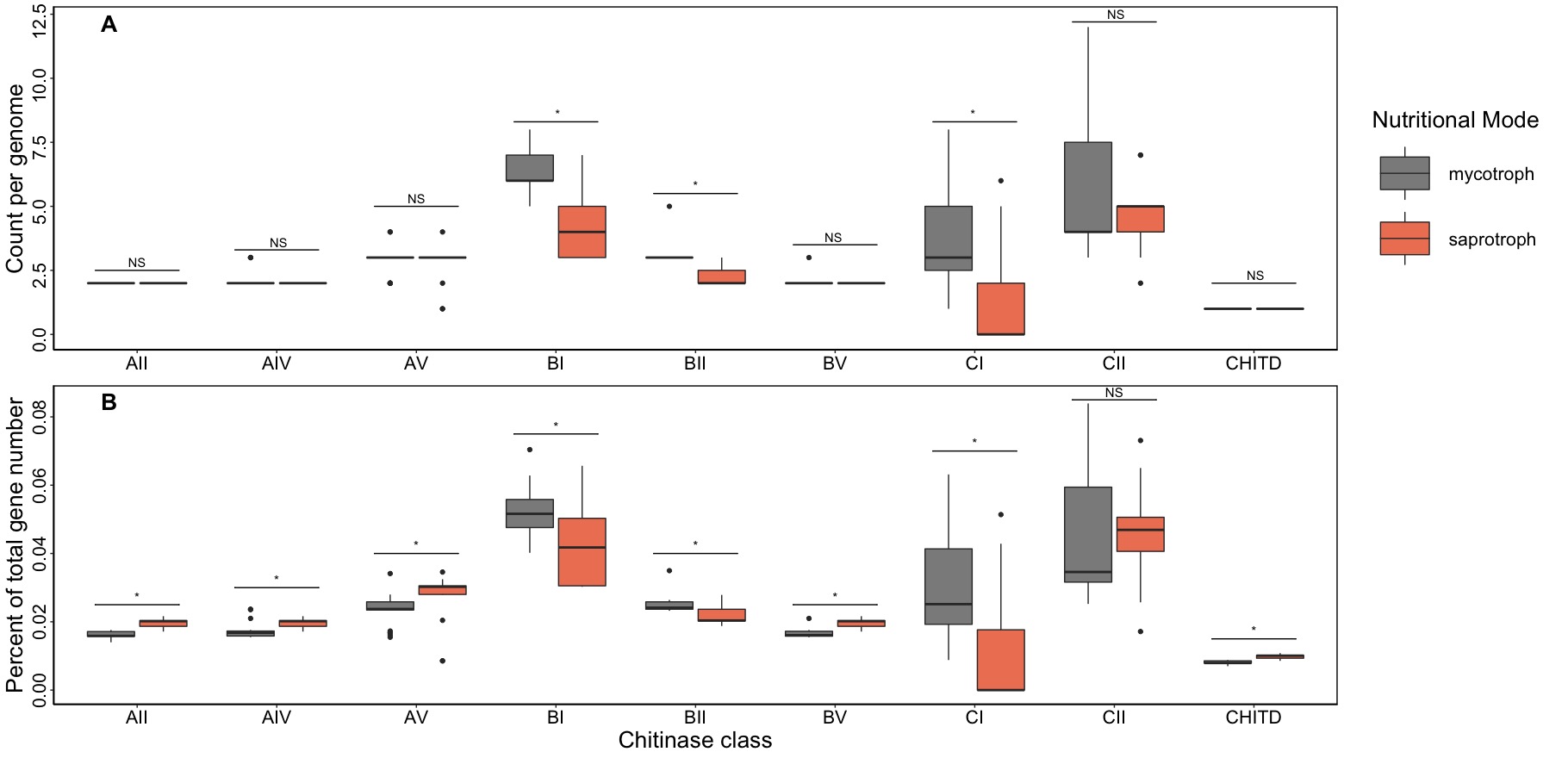

Supplement: S24 Fig — (A) Mycotroph genomes have significantly greater numbers of BI, BII, and CI class chitinase genes compared to saprotroph genomes (2-sample t-test, p ≤ 0.05). (B) Saprotroph genomes have significantly greater percentage of total gene number for AII, AIV, AV, BI, BII, BV, CI, and CHITD class chitinase genes compared to mycotroph genomes (2-sample t-test, p ≤ 0.05). (JPG) [file pone.0289280.s024.jpg]

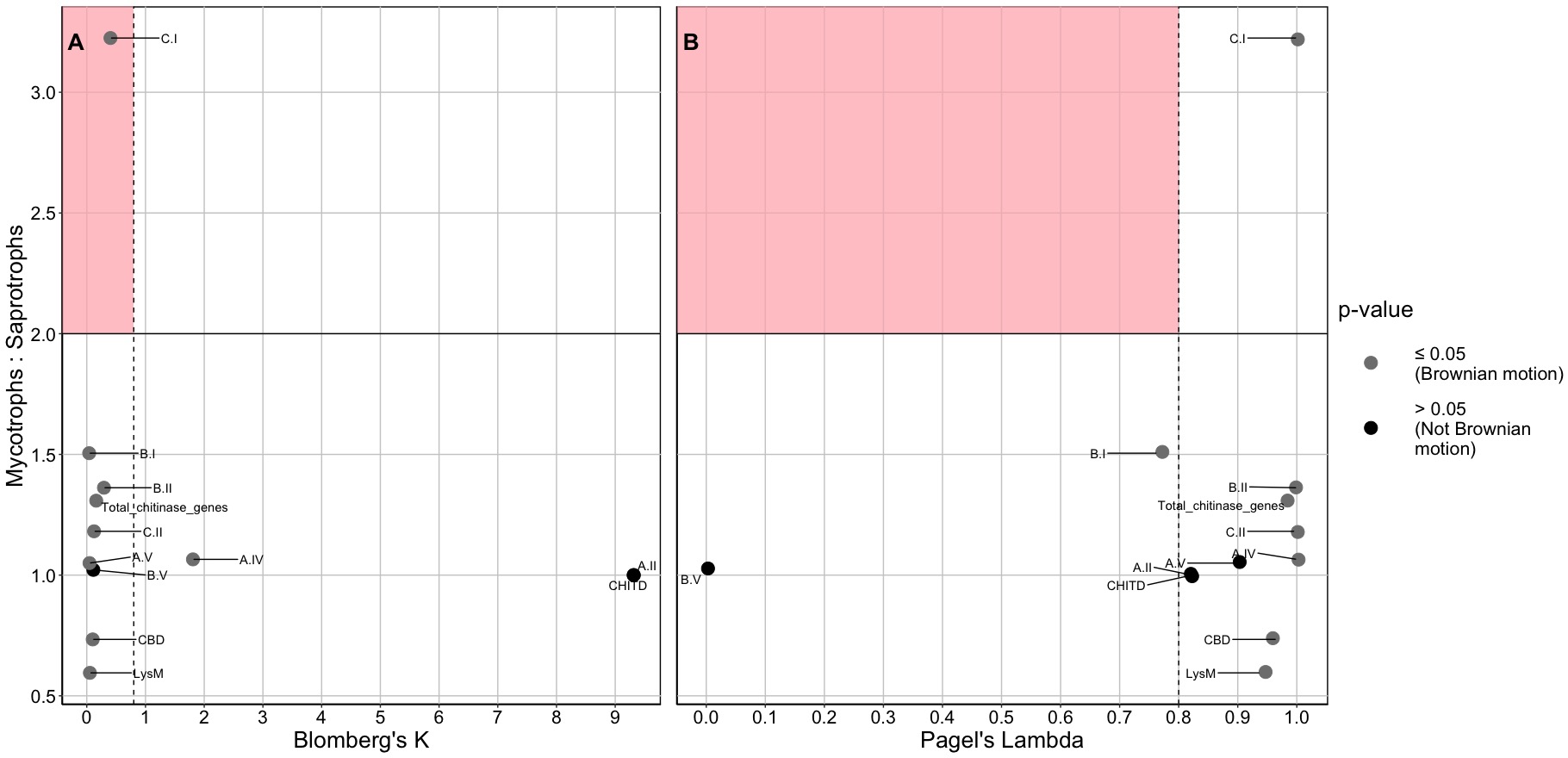

Supplement: S25 Fig — Most evaluated chitinase class distributions were determined to be consistent with Brownian motion evolution as determined by their (A) Blomberg’s K value and significance (p-value ≤ 0.05) and (B) Pagel’s Lambda value and significance (p ≤ 0.05). Black points in the shaded area of the graph would indicate chitinase gene classes which exhibit overdispersal in the Trichoderma phylogeny as well as overrepresentation in mycotroph genomes. (JPG) [file pone.0289280.s025.jpg]

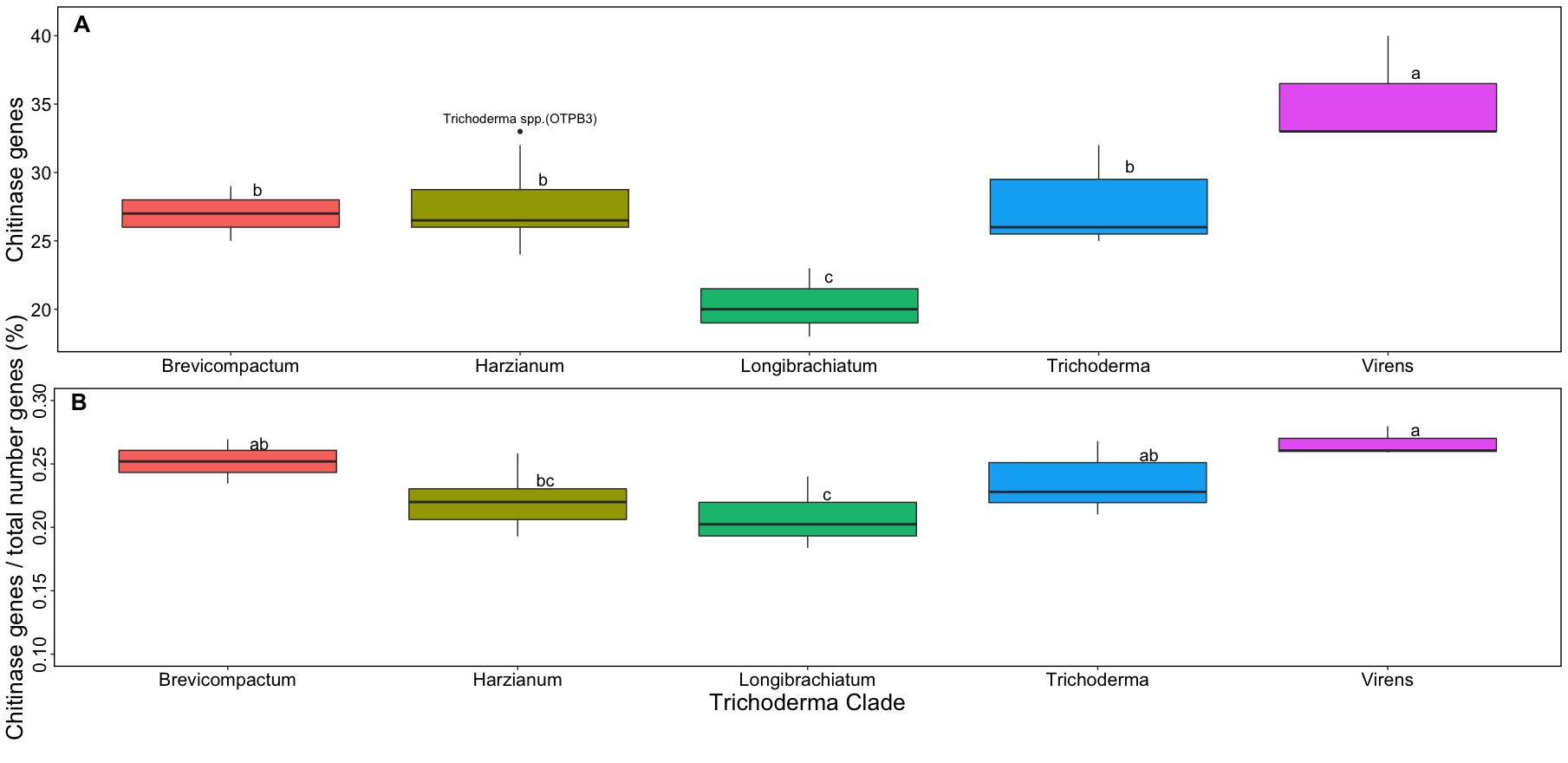

Supplement: S26 Fig — There are significant differences between Trichoderma clades in their (A) number of chitinase genes (p ≤ 0.05) and (B) percent of chitinase genes (p ≤ 0.05). Outlier genomes are labeled. Significant differences are indicated by different letters. Differences were calculated with ANOVA, post hoc tested with Tukey’s HSD. (JPG) [file pone.0289280.s026.jpg]

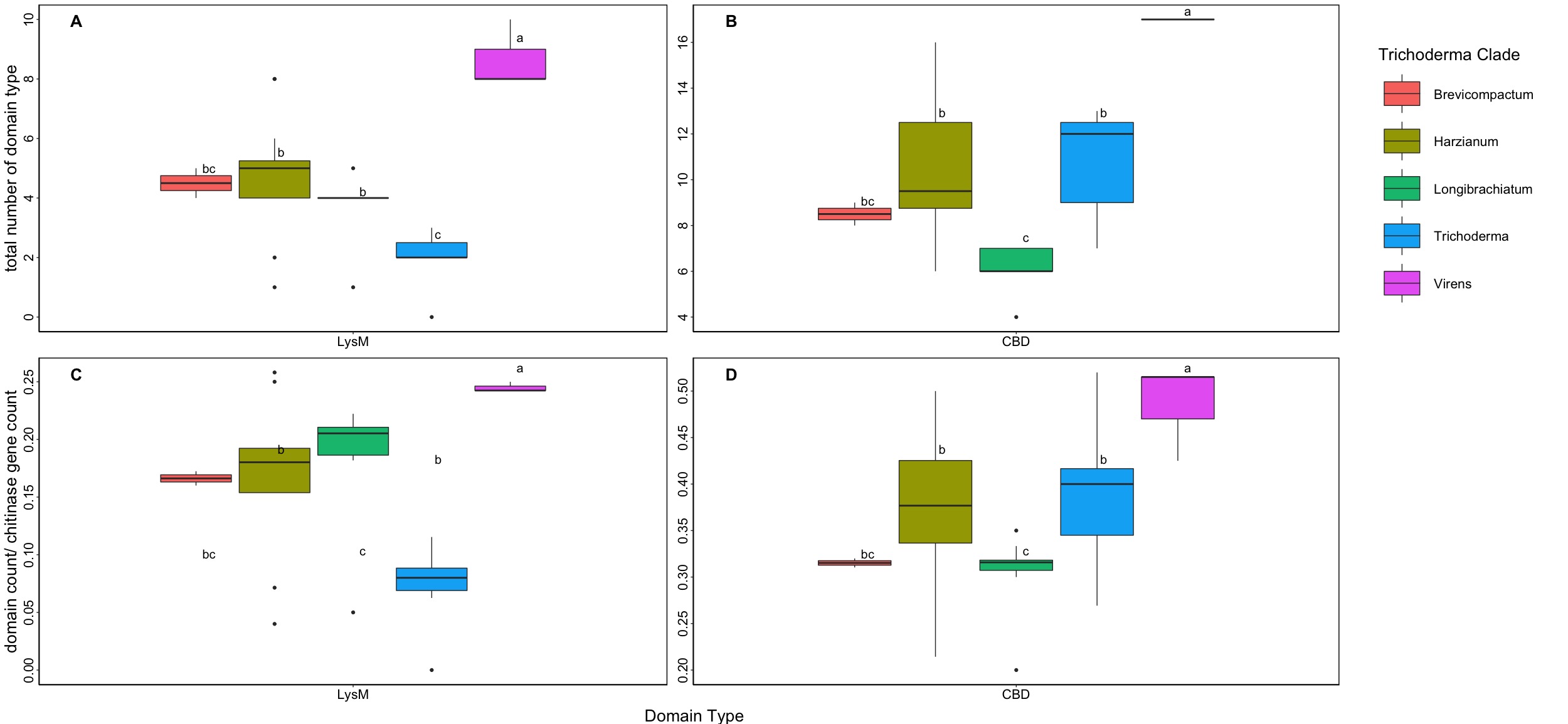

Supplement: S27 Fig — (A) Virens has highest number of LysM and section Trichoderma has the fewest (ANOVA, corrected with Holm-Bonferroni, p ≤ 0.05). (B) Virens has highest number of CBD; Brevicompactum and section Trichoderma have the fewest (ANOVA, corrected with Holm-Bonferroni, p ≤ 0.05). (C) Section Trichoderma has the lowest percentage of LysM to total gene count (ANOVA, corrected with Holm-Bonferroni, p ≤ 0.05). (D) Section Trichoderma has the lowest percentage of CBD to total gene count (ANOVA, corrected with Holm-Bonferroni, p ≤ 0.05). Significant differences are indicated by different letters, as determined by Tukey’s HSD. (JPG) [file pone.0289280.s027.jpg]

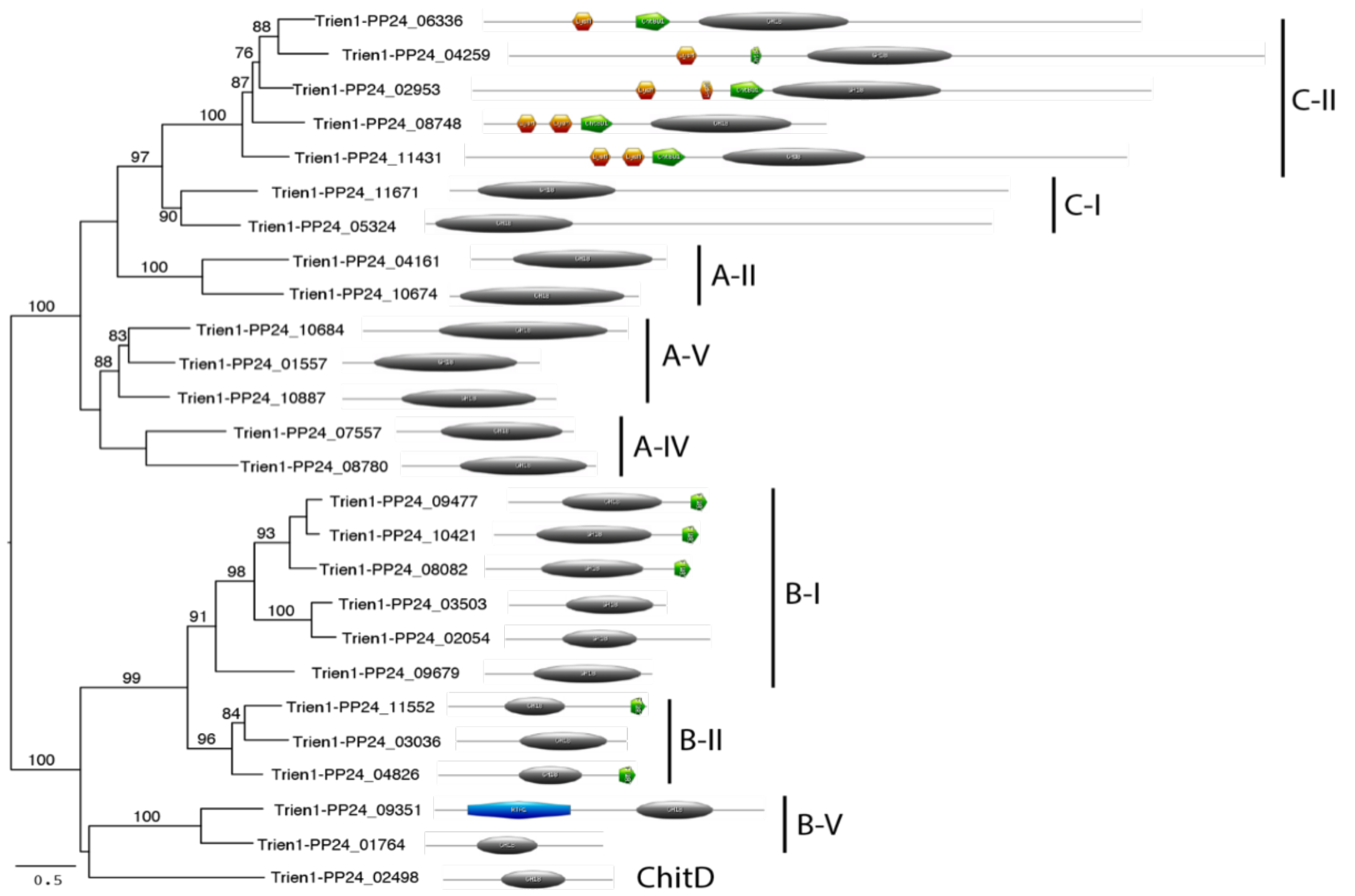

Supplement: S28 Fig — Chitinase classes are indicated according to (Goughenour 2020). GH-18 domains are represented by black ovals, chitin-binding domains (CBD) are represented as green pentagons, Lysin domains (LysM) are represented as orange hexagons, and an RTA1 Superfamily domain is represented by a blue cylinder. (PDF) [file pone.0289280.s028.pdf]

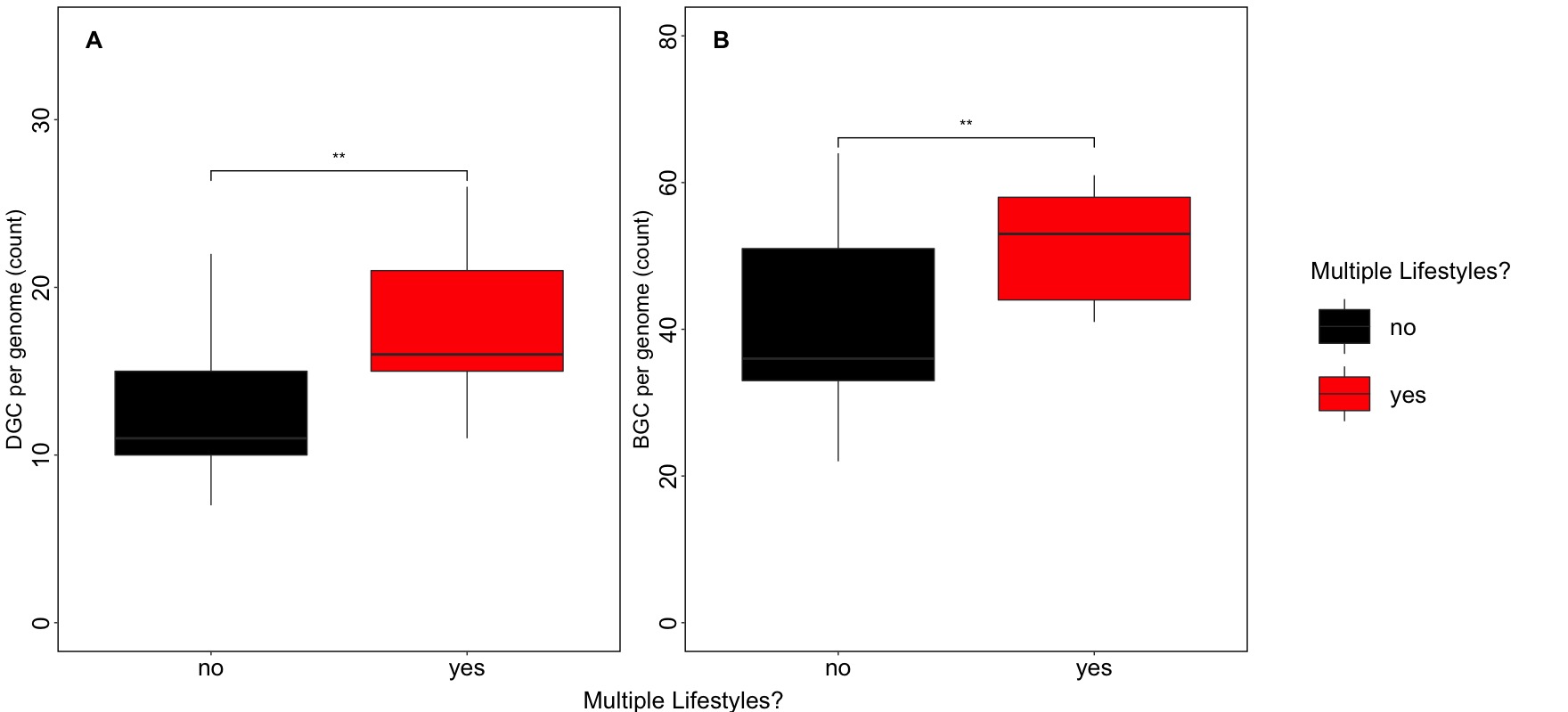

Supplement: S29 Fig — Genomes of Trichoderma species recorded as having multiple lifestyles have (A) a significantly greater diversity of DGC (two-sample t-test, p ≤ 0.05) and (B) a significantly greater number of BGC (two-sample t-test, p ≤ 0.05) as compared to genomes of Trichoderma species recorded as only having a single potential lifestyle. (JPG) [file pone.0289280.s029.jpg]
