# Supplementary material for: Endophyte genomes support greater metabolic gene cluster diversity compared with non-endophytes in Trichoderma
Source: PLoS One. 2023 Dec 21;18(12):e0289280. doi: 10.1371/journal.pone.0289280 (PMC10735191; doi:10.1371/journal.pone.0289280)
Supplement: S3 Fig — (A) The number of DGC families identified in each genome positively correlates with the number of identified biosynthetic gene cluster families (Pearson’s, p < 0.005). (B) The proportion of DGC families to total number genes per genome positively correlates with the proportion of DGC families to total number genes per genome (Pearson’s, p < 0.005). Each point represents a Trichoderma isolate. Point fill indicates nutritional mode, point shape indicates which clade to which the isolate belongs, and color indicates whether or not the isolate has a possible endophytic lifestyle. Gray shaded areas indicate the 95% confidence interval. (PDF) [file pone.0289280.s003.pdf]

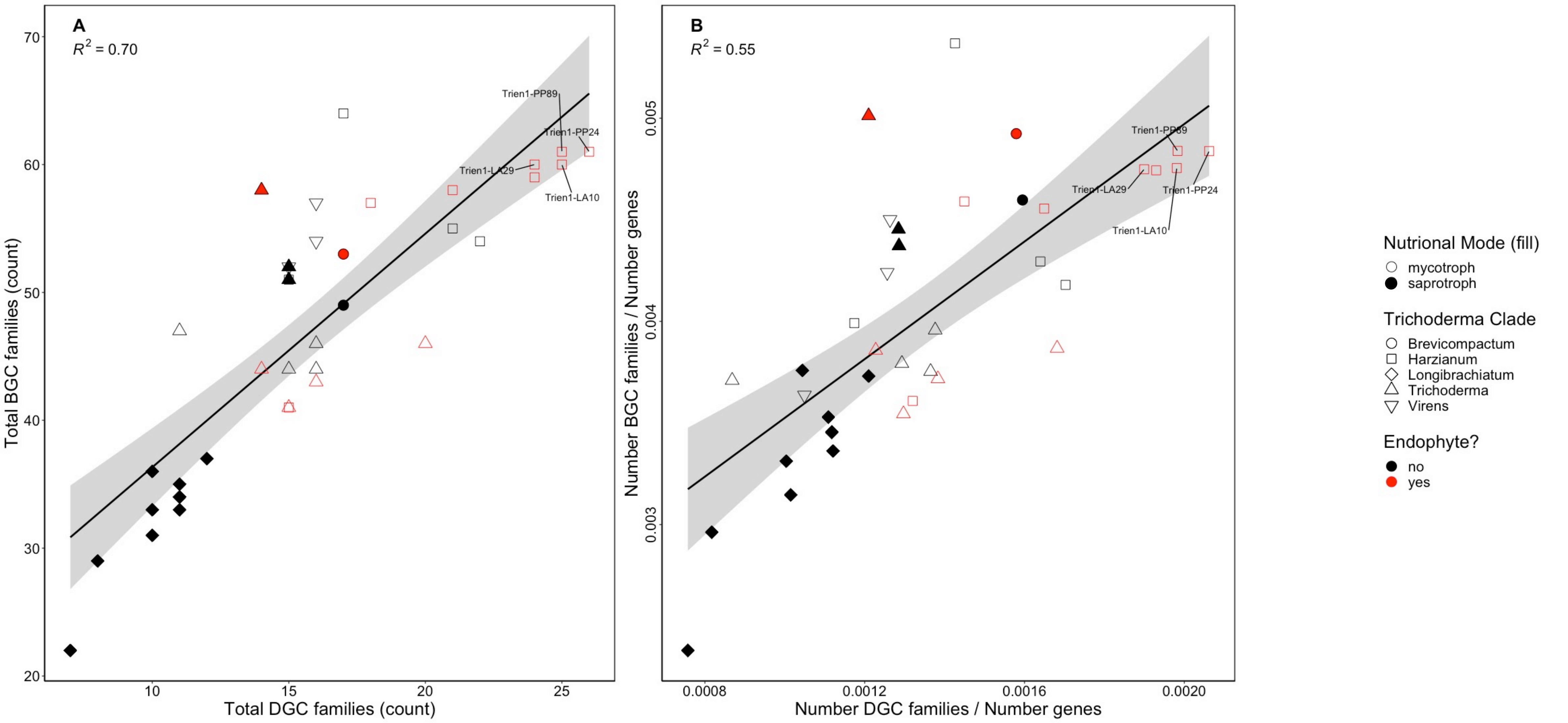
A
R^2 = 0.70
Trien1-PP89
Trien1-PP24
Trien1-LA29
Trien1-LA10
Total BGC families (count)
Total DGC families (count)
B
R^2 = 0.55
Number BGC families / Number genes
Number DGC families / Number genes
Nutrional Mode (fill)
mycotroph
saprotroph
Trichoderma Clade
Brevicompactum
Harzianum
Longibrachiatum
Trichoderma
Virens
Endophyte?
no
yes
