# Supplementary material for: Endophyte genomes support greater metabolic gene cluster diversity compared with non-endophytes in Trichoderma
Source: PLoS One. 2023 Dec 21;18(12):e0289280. doi: 10.1371/journal.pone.0289280 (PMC10735191; doi:10.1371/journal.pone.0289280)
Supplement: S1 Table — (DOCX) [file pone.0289280.s030.docx]

**Table S1. Species name, strain identifier, source of genomic reads, lifestyle information, nutritional mode, and geographic origin of each *Trichoderma* isolate used in this study.**

| **Verified species name** | **Given species name** | **Assembly source (SRA accession)** | **Genome assembly accession** | **Strain** | **Nutritional mode** | **Lifestyle** | **Country of Origin** | **Lifestyle**  **(Endophyte or non-endophyte)** |
| --- | --- | --- | --- | --- | --- | --- | --- | --- |
| *T. afroharzianum** | *T. harzianum* | NCBI | GCA_000988865.1 | T6776 | mycotroph | fungi, soil | Italy | Endophyte |
| *T. afroharzianum** | *T. viride* | NCBI (SRX751293) | This study | LTR-2 | mycotroph | fungi, soil | China | Endophyte |
| *T. arundinaceum* | *T. arundinaceum* | NCBI | GCA_003012105.1 | IBT 40837 | saprotroph | soil | Iran | Non-endophyte |
| *T. asperellum* | *T. asperellum* | NCBI | GCA_000733085.2 | B05 | saprotroph | soil | France | Non-endophyte |
| *T. asperellum* | *T. asperellum* | NCBI | GCA_003025105.1 | CBS 433.97 | saprotroph | soil | United States | Non-endophyte |
| *T. atroviride* | *T. atroviride* | NCBI | GCA_001599035.1 | JCM 9410 | mycotroph | soil, wood/litter | Japan | Non-endophyte |
| *T. atroviride* | *T. atroviride* | NCBI | GCA_002916895.1 | LY357 | mycotroph | soil, wood/litter | China | Non-endophyte |
| *T. atroviride* | *T. atroviride* | NCBI | GCA_000963795.1 | XS2015 | mycotroph | soil, wood/litter | The Netherlands | Non-endophyte |
| *T. atroviride* | *T. atroviride* | NCBI | GCA_000171015.2 | IMI 206040 | mycotroph | soil, wood/litter | Sweden | Non-endophyte |
| *T. bissettii** | *T. koningii* | NCBI | GCA_001950475.1 | JCM 1883 | saprotroph | only known from a human sinus cavity | Wales | Non-endophyte |
| *T. brevicompactum* | *T. brevicompactum* | NCBI | GCA_003012085.1 | IBT 40841 | saprotroph | endophyte, soil | Iran | Endophyte |
| *T. cf. atroviride* | *T. cf. atroviride* | NCBI (SRX1601956) | This study | LU132 | mycotroph | soil, wood/litter | New Zealand | Non-endophyte |
| *T. cf. atroviride* | *T. cf. atroviride* | NCBI (SRX1605616) | This study | LU140 | mycotroph | soil, wood/litter | New Zealand | Non-endophyte |
| *T. citrinoviride* | *T. citrinoviride* | NCBI | GCA_003025115.1 | TUCIM 6016 | saprotroph | wood/litter | Unknown | Non-endophyte |
| *T. endophyticum* | *T. endophyticum* | **This study** | This study | LA10 | mycotroph | endophyte, soil | Peru | Endophyte |
| *T. endophyticum* | *T. endophyticum* | **This study** | This study | LA29 | mycotroph | endophyte, soil | Peru | Endophyte |
| *T. endophyticum* | *T. endophyticum* | **This study** | This study | PP24 | mycotroph | endophyte, soil | Peru | Endophyte |
| *T. endophyticum* | *T. endophyticum* | **This study** | This study | PP89 | mycotroph | endophyte, soil | Peru | Endophyte |
| *T. gamsii* | *T. gamsii* | NCBI | GCA_002894205.1 | A5MH | mycotroph | endophyte, soil | Australia | Endophyte |
| *T. gamsii* | *T. gamsii* | NCBI | GCA_001481775.2 | T6085 | mycotroph | endophyte, soil | Ukraine | Endophyte |
| *T. guizhouense* | *T. guizhouense* | NCBI | GCA_002022785.1 | NJAU 4742 | mycotroph | fungi, wood/litter | China | Endophyte |
| *T. hamatum* | *T. hamatum* | NCBI | GCA_000331835.2 | GD12 | saprotroph | soil | England | Endophyte |
| *T. harzianum* | *T. harzianum* | NCBI | GCA_001990665.1 | B97 | mycotroph | soil | France | Non-endophyte |
| *T. harzianum* | *T. harzianum* | NCBI | GCA_003025095.1 | CBS 226.95 | mycotroph | soil | England | Non-endophyte |
| *T. koningiopsis* | *T. koningiopsis* | NCBI | GCA_002246955.1 | POS7 | mycotroph | endophyte, soil | Argentina | Endophyte |
| *T. longibrachiatum* | *T. longibrachiatum* | NCBI | GCA_003025155.1 | ATCC 18648 | saprotroph | soil | United States | Non-endophyte |
| *T. longibrachiatum* | *T. longibrachiatum* | NCBI | GCA_000332775.1 | SMF2 | saprotroph | soil | China | Non-endophyte |
| *T. parareesei* | *T. parareesei* | NCBI | GCA_001050175.1 | CBS 125925 | saprotroph | wood/litter | Argentina | Non-endophyte |
| *T. pleuroticola** | *T. harzianum* | NCBI | GCA_002894145.1 | Tr1 | mycotroph | fungi | China | Non-endophyte |
| *T. reesei* | *T. reesei* | NCBI | GCA_001999515.1 | CBS 999.97 | saprotroph | wood/litter | French Guiana | Non-endophyte |
| *T. reesei* | *T. reesei* | NCBI | GCA_002006585.1 | QM6a | saprotroph | wood/litter | Solomon Islands | Non-endophyte |
| *T. reesei* | *T. reesei* | NCBI (SRX059777) | This study | QM 9136 | saprotroph | wood/litter | Mutant derived from QM6a | Non-endophyte |
| *T. reesei* | *T. reesei* | NCBI (SRX060131) | This study | QM 9978 | saprotroph | wood/litter | Mutant derived from QM6a | Non-endophyte |
| *T. reesei* | *T. reesei* | NCBI | GCA_000513815.1 | RUT C30 | saprotroph | wood/litter | United States | Non-endophyte |
| *T. simmonsii** | *T. virens* | NCBI | GCA_001931985.1 | IMV 00454 | mycotroph | fungi, wood/litter | Ukraine | Endophyte |
| *T. virens* | *T. virens* | NCBI | GCA_000800515.1 | FT 333 | mycotroph | fungi, soil, wood/litter | Taiwan | Non-endophyte |
| *T. virens* | *T. virens* | NCBI | GCA_001835465.1 | IMI 304061 | mycotroph | fungi, soil, wood/litter | India | Non-endophyte |
| *T. virens* | *T. virens* | NCBI | GCA_000170995.2 | Gv29.8 | mycotroph | fungi, soil, wood/litter | Unknown | Non-endophyte |
| *Trichoderma sp.** | *T. harzianum* | NCBI (SRX1433332) | This study | OTPB3 | mycotroph | unknown | India | Non-endophyte |

*Some isolates were previously identified as different species but were re-identified for this study.
