# Supplementary material for: Endophyte genomes support greater metabolic gene cluster diversity compared with non-endophytes in Trichoderma
Source: PLoS One. 2023 Dec 21;18(12):e0289280. doi: 10.1371/journal.pone.0289280 (PMC10735191; doi:10.1371/journal.pone.0289280)
Supplement: S2 Table — (DOCX) [file pone.0289280.s031.docx]

**Table S2. Genome statistics of the 39 *Trichoderma* isolates.**

| **Verified species name** | **Strain** | **Complete and single-copy BUSCOs (%)** | **Number genes (post-Orthofiller)** | **N50** | **Genome length (Mb)** |
| --- | --- | --- | --- | --- | --- |
| *T. arundinaceum* | IBT 40837 | 96.7 | 10,658 | 61,935 | 36.9 |
| *T. asperellum* | B05 | 96.4 | 11,676 | 99,299 | 37.7 |
| *T. asperellum* | CBS 433.97 | 98.8 | 11,666 | 2,128,412 | 37.5 |
| *T. atroviride* | JCM 9410 | 98.9 | 11,600 | 5,619,901 | 37.3 |
| *T. cf. atroviride* | LU132 | 72.7 | 12,669 | 9,580 | 35.4 |
| *T. cf. atroviride* | LU140 | 61.2 | 8,243 | 5,723 | 33.7 |
| *T. atroviride* | LY357 | 98.5 | 11,565 | 100,133 | 35.9 |
| *T. atroviride* | XS2015 | 98.9 | 11,722 | 2,105,403 | 36.4 |
| *T. atroviride* | IMI 206040 | 98.7 | 11,624 | 2,007,903 | 36.1 |
| *T. brevicompactum* | IBT 40841 | 97.4 | 10,764 | 58,500 | 37.0 |
| *T. citrinoviride* | TUCIM 6016 | 95.5 | 9,918 | 1,846,965 | 33.2 |
| *T. endophyticum* | LA10 | 99.0 | 12,619 | 1,095,838 | 39.2 |
| *T. endophyticum* | LA29 | 99.0 | 12,635 | 1,266,890 | 39.2 |
| *T. endophyticum* | PP24 | 99.0 | 12,608 | 713,317 | 38.9 |
| *T. endophyticum* | PP89 | 99.1 | 12,606 | 1,414,947 | 38.9 |
| *T. gamsii* | A5MH | 98.7 | 11,567 | 594,470 | 38.5 |
| *T. gamsii* | T6085 | 98.8 | 11,893 | 697,391 | 37.9 |
| *T. guizhouense* | NJAU 4742 | 98.1 | 12,438 | 2,414,983 | 38.3 |
| *T. harzianum* | B97 | 98.3 | 12,806 | 113,316 | 40.7 |
| *T. harzianum* | CBS 226.95 | 98.8 | 12,920 | 2,414,909 | 41.0 |
| *Trichoderma sp.* | OTPB3 | 98.1 | 12,777 | 142,666 | 42.0 |
| *T. afroharzianum* | T6776 | 98.0 | 12,418 | 68,846 | 39.7 |
| *T. pleuroticola* | Tr1 | 98.1 | 11,921 | 626,242 | 38.8 |
| *T. hamatum* | GD12 | 97.9 | 11,572 | 179,274 | 38.4 |
| *T. bissettii* | JCM 1883 | 98.5 | 9,242 | 4,316,785 | 32.3 |
| *T. koningiopsis* | POS7 | 97.4 | 11,405 | 1,749,579 | 36.6 |
| *T. longibrachiatum* | ATCC 18648 | 87.5 | 9,789 | 1,606,350 | 32.2 |
| *T. longibrachiatum* | SMF2 | 98.4 | 9,854 | 863,071 | 31.7 |
| *T. parareesei* | CBS 125925 | 97.1 | 9,578 | 68,608 | 32.1 |
| *T. reesei* | CBS 999.97 | 98.6 | 9,844 | 1,217,941 | 32.5 |
| *T. reesei* | QM6a | 99.0 | 9,839 | 5,311,445 | 34.9 |
| *T. reesei* | QM 9136 | 97.8 | 9,963 | 60,397 | 32.6 |
| *T. reesei* | QM 9978 | 97.9 | 9,918 | 61,591 | 33.0 |
| *T. reesei* | RUT C30 | 98.8 | 9,815 | 1,023,047 | 32.7 |
| *T. virens* | FT 333 | 98.1 | 12,734 | 173,918 | 38.6 |
| *T. virens* | IMI 304061 | 98.8 | 14,297 | 722,602 | 45.8 |
| *T. afroharzianum* | LTR-2 | 98.8 | 11,362 | 92,065 | 38.2 |
| *T. simmonsii* | IMV 00454 | 98.5 | 12,733 | 1,319,489 | 42.0 |
| *T. virens* | Gv29.8 | 99.0 | 12,664 | 1,836,662 | 39.0 |
