# Supplementary material for: Endophyte genomes support greater metabolic gene cluster diversity compared with non-endophytes in Trichoderma
Source: PLoS One. 2023 Dec 21;18(12):e0289280. doi: 10.1371/journal.pone.0289280 (PMC10735191; doi:10.1371/journal.pone.0289280)
Supplement: S3 Table — (DOCX) [file pone.0289280.s032.docx]

**Table S3. Mycoparasitism gene orthogroups of interest found in the highest endophyte:non-endophyte ratio.**

| Mycoparasitism Gene orthogroup | Pagel’s Lambda | Pagel’s Lambda p-value | Blomberg’s K | Blomberg’s K p-value | M:S* | E:NE** | Eggnog Annotation |
| --- | --- | --- | --- | --- | --- | --- | --- |
| OG0010098 | 0.999 | 8.71E-12 | 0.325 | 0.001 | 9.130 | 4.714 | Unknown Function |
| OG0000661 | 0.917 | 1.11E-08 | 0.334 | 0.001 | 4.099 | 3.143 | Function unknown (NAD(P)H-binding) |
| OG0009104 | 0.921 | 5.62E-08 | **0.0140** | **0.082** | 4.783 | 3.048 | Unknown Function |
| OG0010147 | 0.999 | 2.38E-17 | 0.471 | 0.001 | 4.565 | 2.857 | Function unknown (Zinc Finger) |
| OG0009580 | 0.999 | 2.50E-30 | 2.99 | 0.001 | 13.043 | 2.786 | Post-translational modification, protein turnover, and chaperones (Eukaryotic aspartyl protease) |
| OG0009038 | 0.999 | 2.16E-10 | 0.230 | 0.002 | 3.587 | 2.743 | Unknown Function |
| OG0009716 | 0.999 | 7.65E-20 | 0.817 | 0.001 | 3.261 | 2.694 | Unknown Function |
| OG0008134 | 0.961 | 1.59E-07 | **0.004** | **0.284** | 6.522 | 2.637 | Unknown Function (X-Pro dipeptidyl-peptidase C-terminal non-catalytic domain) |
| OG0010671 | 0.780 | 0.00094367 | 0.0324 | 0.02 | 1.304 | 2.400 | Unknown Function |
| OG0009627 | **0.161** | **0.211** | 0.039 | 0.009 | 2.446 | 2.357 | Unknown Function |

*Ratio of gene count in mycotroph to saprotroph *Trichoderma* genomes

**Ratio of gene count in endophytic to non-endophytic *Trichoderma* genomes
