# Supplementary material for: Endophyte genomes support greater metabolic gene cluster diversity compared with non-endophytes in Trichoderma
Source: PLoS One. 2023 Dec 21;18(12):e0289280. doi: 10.1371/journal.pone.0289280 (PMC10735191; doi:10.1371/journal.pone.0289280)
Supplement: S4 Table — (DOCX) [file pone.0289280.s033.docx]

**Table S4. Mycoparasitism gene orthogroups of interest found in the highest mycotroph:saprotroph ratios as well as orthogroups only found in mycotrophs.**

| Mycoparasitism Gene orthogroup | Pagel’s Lambda | Pagel’s Lambda p-value | Blomberg’s K | Blomberg’s K p-value | M:S* | E:NE** | Eggnog Annotation |
| --- | --- | --- | --- | --- | --- | --- | --- |
| OG0008901 | 0.99 | 7.14E-24 | 1.029 | 0.001 | --*** | 1.978 | Amino acid transport and metabolism (Amino acid transport and metabolism) |
| OG0009237 | 0.99 | 1.23E-21 | 0.830 | 0.001 | -- | 1.714 | Unknown Function (LamB/YcsF family) |
| OG0009490 | 0.999 | 4.45E-28 | 1.997 | 0.001 | -- | 1.558 | Unknown Function (spectrin binding) |
| OG0010126 | 0.999 | 1.31E-23 | 0.93 | 0.001 | -- | 1.714 | Unknown Function |
| OG0010265 | 0.999 | 4.53E-41 | 8.061 | 0.001 | -- | 1.959 | Carbohydrate transport and metabolism (glycerone kinase activity) |
| OG0010647 | 0.999 | 3.14E-14 | 0.304 | 0.001 | -- | 2.000 | Unknown Function (Domain of unknown function DUF3328) |
| OG0011909 | 0.999 | 1.12E-24 | 1.617 | 0.001 | -- | 1.286 | No orthologs found |
| OG0011914 | 0.817 | 0.00596218 | 0.050 | 0.031 | -- | 0.686 | Unknown Function |
| OG0012676 | 0.999 | 5.02E-23 | 1.307 | 0.001 | -- | 0.429 | Unknown Function |
| OG0012734 | 0.999 | 5.02E-23 | 1.307 | 0.001 | -- | 0.429 | Nucleotide transport and metabolism, Translation, ribosomal structure and biogenesis (phospholipid biosynthetic process) |
| OG0013241 | 0.999 | 8.90E-15 | 0.410 | 0.001 | -- | 0.000 | Unknown Function (zinc finger) |
| OG0014884 | 0.999 | 2.87E-09 | 0.252 | 0.016 | -- | 0.000 | Unknown Function (Heterokaryon incompatibility protein (HET) |
| OG0016824 | **0.096** | **0.35427001** | **0.020** | **0.383** | -- | 0.000 | Post-translational modification, protein turnover, and chaperones (Belongs to the peptidase C1 family) |
| OG0009580 | 0.999 | 2.50E-30 | 2.99 | 0.001 | 13.043 | 2.786 | Post-translational modification, protein turnover, and chaperones (Eukaryotic aspartyl protease) |
| OG0009990 | 0.999 | 5.95E-22 | 0.848 | 0.001 | 10.434 | 1.929 | Unknown Function |
| OG0010098 | 0.999 | 8.71E-12 | 0.325 | 0.001 | 9.130 | 4.714 | Unknown Function |
| OG0002822 | 0.999 | 1.34E-19 | 0.68 | 0.001 | 7.826 | 2.000 | Unknown Function (Serine hydrolase) (FSH1) |
| OG0009146 | 0.999 | 7.54E-11 | 0.224 | 0.001 | 7.500 | 1.857 | Translation, ribosomal structure and biogenesis (amidase C869.01) |
| OG0008134 | 0.961 | 1.59E-07 | **0.004** | **0.284** | 6.521 | 2.63 | Unknown Function (X-Pro dipeptidyl-peptidase C-terminal non-catalytic domain) |
| OG0009419 | 0.999 | 9.95E-23 | 1.014 | 0.001 | 6.522 | 1.714 | Carbohydrate transport and metabolism, Cell wall/membrane/envelope biogenesis (NmrA-like family) |
| OG0008747 | 0.564 | 0.00033682 | 0.267 | 0.001 | 5.652 | 2.110 | Function unknown (TAP-like protein) |
| OG0008746 | 0.99 | 1.70E-14 | 0.307 | 0.001 | 5.435 | 1.486 | Unknown Function |
| OG0011291 | 0.747 | 2.51E-06 | 0.01 | 0.167 | 5.217 | 1.371 | Unknown Function |
| OG0011298 | 0.999 | 3.12E-05 | 0.12 | 0.002 | 5.217 | 2.143 | Unknown Function |

*Ratio of gene count in mycotrophic to saprotrophic *Trichoderma* genomes

**Ratio of gene count in endophytic to non-endophytic *Trichoderma* genomes

***Gene orthogroups that were not detected in saprotrophic genomes are designated with a “--” in place of a mycotroph:saprotroph ratio.
