# Supplementary material for: Endophyte genomes support greater metabolic gene cluster diversity compared with non-endophytes in Trichoderma
Source: PLoS One. 2023 Dec 21;18(12):e0289280. doi: 10.1371/journal.pone.0289280 (PMC10735191; doi:10.1371/journal.pone.0289280)
Supplement: S5 Table — (DOCX) [file pone.0289280.s034.docx]

**Table S5. Placement of Brevicompactum clade ergot alkaloid BGC gene phylogenies**

| **Gene** | **In clade of predominantly Hypocreales spp.** | **In clade of predominantly Xylariales spp.** | **Node support** | **Noteworthy results** |
| --- | --- | --- | --- | --- |
| Tribre1_123771 elymoclavine monooxygenase (*cloA*) | X | - | 100 | Brevicompactum clade is sister to other Hypocreales |
| Tribre1_123772 Chanoclavine synthase catalase (*easC*) | - | - |  | Other *Trichoderma* in a clade with a homolog of tribre1 query gene |
| Tribre1_123773 Lysergyl peptide synthetase subunit 2 (*lps2*) | - | - |  |  |
| Tribre1_123774 chanoclavine-I dehydrogenase (*easD)* | - | X | 96 | aspcor2, psevol1 within clade |
| Tribre1_123775 Chanoclavine-I aldehyde oxidoreductase (*easA*) | - | X | 100 | aspcor2, psevol1 within clade, Pleosporales in outgroup |
| Tribre1_123776 argoclavine dehydrogenase (*easG)* | - | X | 95 | aspcor2 sister to clade |
| Tribre1_123777 chanoclavine-I synthase oxidoreductase (*easE*) | - | X | 100 | aspcor2, psevol1 within clade |
| Dimethylallyltryptophan N-methyltransferase (*easF*) | - | X | 100 | aspcor2, psevol1 within clade |
| Tribre1_123778 Dimethylallyltryptophan synthase (*dmaW*) | - | X | 100 | aspcor2 within clade; bipsor1.1, cocsat1 (Pleosporales) in clade. Pleosporales have 100% support with Xylariales sp. (mictri1) |
| Tribre1_123779 hypothetical protein | - | X | 100 | Absent from Clavicipitaceae, largely recovered in Xylariales, other Trichoderma in the sameclade, 1 copy/genome |
| Tribre1_123780 oxygenase (*easH*) | - | X | 100 | aspcor2, parnod1.1, epifes1.1 in clade |
| Tribre1_123781 Lysergyl peptide synthetase subunit 1 (*lps1*) | - | X | 52 | aspcor2, epifes1.1 in clade |
