# Supplementary material for: Endophyte genomes support greater metabolic gene cluster diversity compared with non-endophytes in Trichoderma
Source: PLoS One. 2023 Dec 21;18(12):e0289280. doi: 10.1371/journal.pone.0289280 (PMC10735191; doi:10.1371/journal.pone.0289280)
Supplement: S6 Table — (DOCX) [file pone.0289280.s035.docx]

**Table S6**. **Functional annotations of the top 21 mycoparasitism genes contributing to the global PC1 and PC2 in the pPCA analysis.**

| **Orthogroup** | **Functional Category** | **Description** |
| --- | --- | --- |
| OG0000020 | Secondary metabolites biosynthesis, transport, and catabolism | Enoyl-(Acyl carrier protein) reductase |
| OG0009047 | Function unknown | -- |
| OG0008954 | Function unknown | oligopeptide transmembrane transporter activity |
| OG0009301 | Function unknown | -- |
| OG0008688 | Translation, ribosomal structure and biogenesis | maturation of LSU-rRNA |
| OG0000599 | Function unknown | -- |
| OG0008901 | Amino acid transport and metabolism | glutamine biosynthetic process |
| OG0000008 | Secondary metabolites biosynthesis, transport, and catabolism | phosphopantetheine binding |
| OG0000642 | Post-translational modification, protein turnover, and chaperones | Belongs to the peptidase S1 family |
| OG0010265 | Carbohydrate transport and metabolism | glycerone kinase activity |
| OG0011331 | Defense mechanisms | ATPase activity |
| OG0011314 | Function unknown | -- |
| OG0011345 | Function unknown | -- |
| OG0008929 | Function unknown | -- |
| OG0008903 | Inorganic ion transport and metabolism | alkaline phosphatase activity |
| OG0008978 | Function unknown | trichodiene synthase activity |
| OG0001105 | Inorganic ion transport and metabolism | O-methyltransferase activity |
| OG0001172 | Energy production and conversion | alpha-methylacyl-CoA racemase activity |
| OG0000193 | Defense mechanisms | ATPase activity |
| OG0000045 | Function unknown | -- |
| OG0000267 | Function unknown | -- |
