# Supplementary material for: Endophyte genomes support greater metabolic gene cluster diversity compared with non-endophytes in Trichoderma
Source: PLoS One. 2023 Dec 21;18(12):e0289280. doi: 10.1371/journal.pone.0289280 (PMC10735191; doi:10.1371/journal.pone.0289280)
Supplement: S7 Table — (DOCX) [file pone.0289280.s036.docx]

**Table S7. EasA phylogenies for the consensus phylogeny and the forced monophyletic phylogenies for Xylariales, Hypocreales, and Eurotiales.**

| **Tree** | **logL** | **deltaL** | **bp-RELL** | **p-KH** | **p-SH** | **c-ELW** | **p-AU** |
| --- | --- | --- | --- | --- | --- | --- | --- |
| Xylariales monophyly^*^ | -22823.99931 | 10280 | 0 | 0 | 0 | 0 | 2.42E-33 |
| Hypocreales monophyly^†^ | -21849.71413 | 9305.3 | 0 | 0 | 0 | 0 | 5.42E-68 |
| Eurotiales monophyly^‡^ | -23882.56281 | 11338 | 0 | 0 | 0 | 0 | 1.16E-85 |
| Consensus tree^§^ | -12544.45575 | 0 | 1 | 1 | 1 | 1 | 1 |

*((anntru1_122141,monsp.4_RYP93154.1,dalchi1_KAF3063816.1,daldec1_30240,dalloc2_490045,dalloc3_108807,xylnig1_360855,xyltel1_247880,monibe1_RYP10946.1,hypmon1_288472,hypsp.10_85717,hypsp.8_352432,hypsp.5_646361,dalbam1_532614,dalesc1_384250,dalesc2_368012,dalsp.2_OTB14163.1,dalver1_236342),(aspcor2_158399,aspfum3_3266,aspfum6.1_EDP55299.1,aspfum5.1_EAL94095.1,aspfum7_KEY80397.1,aspfum4_KMK61248.1,asphan1.1_KAF7593453.1,asplep1_197581,aspind2_391541,penroq1_4309,bysspe1_6999,paevar3_GAD93026.1,glaloz1_3738,claaff1.1_KAG6063203.1,claaru1_KAG5965186.1,clahum1.1_KAG6117434.1,claaru2.1_KAG5953672.1,claaff2_KAG6298797.1,clapur1_2814,clamon1_KAG5937939.1,clacap1_KAG5919219.1,clapaz1_KAG5934472.1,clacyp1_KAG5963712.1,clasp.10_KAG6074294.1,clasp.3_KAG6056451.1,clasp.6_KAG6075262.1,clasp.11_KAG6097320.1,clasp.7_KAG6113323.1,clasp.2_KAG6081721.1,clasp.8_KAG6112740.1,claspa1_KAG5987848.1,clasp.4_KAG6033495.1,clasp.5_KAG6047856.1,clasp.9_KAG6027763.1,claafr1.1_KAG5924161.1,clasor1.1_KAG5918133.1,cladig1.1_KAG5979913.1,clalov1.1_KAG5999390.1,clapus1.1_KAG5986315.1,metacr1_6981,metani1_9795,metani2_KJK79258.1,metani3_KJK95579.1,methum1_KAH0593516.1,metrob2.1_EXU97505.1,metrob4_mtdb2590,metrob3.1_EFY97960.1,metbru1_4220,metgui1_9983,metalb1_1234,epifes1.1_QPG95279.1,asphan1.1_KAF7588053.1,asplep1_157608,asphom1_468681,penexp2_712,asplep1_20392,triaru1_6994,tribre1_123775,psevol1.1_KAG9372089.1));

^†^((claaff1.1_KAG6063203.1,claaru1_KAG5965186.1,clahum1.1_KAG6117434.1,claaru2.1_KAG5953672.1,claaff2_KAG6298797.1,clapur1_2814,clamon1_KAG5937939.1,clacap1_KAG5919219.1,clapaz1_KAG5934472.1,clacyp1_KAG5963712.1,clasp.10_KAG6074294.1,clasp.3_KAG6056451.1,clasp.6_KAG6075262.1,clasp.11_KAG6097320.1,clasp.7_KAG6113323.1,clasp.2_KAG6081721.1,clasp.8_KAG6112740.1,claspa1_KAG5987848.1,clasp.4_KAG6033495.1,clasp.5_KAG6047856.1,clasp.9_KAG6027763.1,claafr1.1_KAG5924161.1,clasor1.1_KAG5918133.1,cladig1.1_KAG5979913.1,clalov1.1_KAG5999390.1,clapus1.1_KAG5986315.1,metacr1_6981,metani1_9795,metani2_KJK79258.1,metani3_KJK95579.1,methum1_KAH0593516.1,metrob2.1_EXU97505.1,metrob4_mtdb2590,metrob3.1_EFY97960.1,metbru1_4220,metgui1_9983,metalb1_1234,epifes1.1_QPG95279.1,triaru1_6994,tribre1_123775),(anntru1_122141,aspcor2_158399,aspfum3_3266,aspfum6.1_EDP55299.1,aspfum5.1_EAL94095.1,aspfum7_KEY80397.1,aspfum4_KMK61248.1,asphan1.1_KAF7593453.1,asplep1_197581,aspind2_391541,penroq1_4309,bysspe1_6999,paevar3_GAD93026.1,glaloz1_3738,monsp.4_RYP93154.1,asphan1.1_KAF7588053.1,asplep1_157608,asphom1_468681,penexp2_712,asplep1_20392,dalchi1_KAF3063816.1,daldec1_30240,dalloc2_490045,dalloc3_108807,xylnig1_360855,xyltel1_247880,monibe1_RYP10946.1,psevol1.1_KAG9372089.1,hypmon1_288472,hypsp.10_85717,hypsp.8_352432,hypsp.5_646361,dalbam1_532614,dalesc1_384250,dalesc2_368012,dalsp.2_OTB14163.1,dalver1_236342));

^‡^((aspcor2_158399,aspfum3_3266,aspfum6.1_EDP55299.1,aspfum5.1_EAL94095.1,aspfum7_KEY80397.1,aspfum4_KMK61248.1,asphan1.1_KAF7593453.1,asplep1_197581,aspind2_391541,penroq1_4309,bysspe1_6999,paevar3_GAD93026.1,asphan1.1_KAF7588053.1,asplep1_157608,asphom1_468681,penexp2_712,asplep1_20392,psevol1.1_KAG9372089.1),(anntru1_122141,glaloz1_3738,claaff1.1_KAG6063203.1,claaru1_KAG5965186.1,clahum1.1_KAG6117434.1,claaru2.1_KAG5953672.1,claaff2_KAG6298797.1,clapur1_2814,clamon1_KAG5937939.1,clacap1_KAG5919219.1,clapaz1_KAG5934472.1,clacyp1_KAG5963712.1,clasp.10_KAG6074294.1,clasp.3_KAG6056451.1,clasp.6_KAG6075262.1,clasp.11_KAG6097320.1,clasp.7_KAG6113323.1,clasp.2_KAG6081721.1,clasp.8_KAG6112740.1,claspa1_KAG5987848.1,clasp.4_KAG6033495.1,clasp.5_KAG6047856.1,clasp.9_KAG6027763.1,claafr1.1_KAG5924161.1,clasor1.1_KAG5918133.1,cladig1.1_KAG5979913.1,clalov1.1_KAG5999390.1,clapus1.1_KAG5986315.1,metacr1_6981,metani1_9795,metani2_KJK79258.1,metani3_KJK95579.1,methum1_KAH0593516.1,metrob2.1_EXU97505.1,metrob4_mtdb2590,metrob3.1_EFY97960.1,metbru1_4220,metgui1_9983,metalb1_1234,epifes1.1_QPG95279.1,monsp.4_RYP93154.1,dalchi1_KAF3063816.1,daldec1_30240,dalloc2_490045,dalloc3_108807,triaru1_6994,tribre1_123775,xylnig1_360855,xyltel1_247880,monibe1_RYP10946.1,hypmon1_288472,hypsp.10_85717,hypsp.8_352432,hypsp.5_646361,dalbam1_532614,dalesc1_384250,dalesc2_368012,dalsp.2_OTB14163.1,dalver1_236342));

^§^(anntru1_122141:0.0907767,((((((aspcor2_158399:0.146401,(((((((((((aspfum3_3266:2.2143e-06,aspfum6.1_EDP55299.1:0.00289032)39:2.2143e-06,aspfum5.1_EAL94095.1:2.2143e-06)49:2.2143e-06,aspfum7_KEY80397.1:0.00580054)94:0.00511127,aspfum4_KMK61248.1:0.00789025)100:0.165702,(asphan1.1_KAF7593453.1:0.22098,asplep1_197581:0.0789538)100:0.0851593)99:0.0947185,(aspind2_391541:0.354398,penroq1_4309:0.331419)99:0.108678)98:0.0841025,(bysspe1_6999:2.2143e-06,paevar3_GAD93026.1:2.2143e-06)100:0.294119)100:0.294486,glaloz1_3738:0.50645)86:0.0420959,(((((((claaff1.1_KAG6063203.1:0.00808026,((claaru1_KAG5965186.1:0.00269054,clahum1.1_KAG6117434.1:2.2143e-06)59:2.2143e-06,claaru2.1_KAG5953672.1:0.00268879)98:0.00543)89:0.00821183,(((claaff2_KAG6298797.1:2.2143e-06,clapur1_2814:0.00818494)99:0.00273485,clamon1_KAG5937939.1:0.0221893)92:0.00246982,(clacap1_KAG5919219.1:0.0109145,clapaz1_KAG5934472.1:0.0054326)100:0.0111715)96:0.011037)38:2.1127e-06,((clacyp1_KAG5963712.1:0.034325,(clasp.10_KAG6074294.1:2.2143e-06,(clasp.3_KAG6056451.1:2.2143e-06,clasp.6_KAG6075262.1:2.2143e-06)100:0.0027152)100:0.0294291)80:0.00166618,((((clasp.11_KAG6097320.1:2.2143e-06,clasp.7_KAG6113323.1:2.2143e-06)50:2.2143e-06,clasp.2_KAG6081721.1:0.00269603)100:0.0109122,clasp.8_KAG6112740.1:0.00268378)98:0.00268724,claspa1_KAG5987848.1:2.2143e-06)96:0.00271401)63:0.00270971)82:0.000771303,(clasp.4_KAG6033495.1:2.2143e-06,(clasp.5_KAG6047856.1:0.00268121,clasp.9_KAG6027763.1:0.00811599)63:2.2143e-06)99:0.012759)100:0.129223,((claafr1.1_KAG5924161.1:0.0780362,clasor1.1_KAG5918133.1:0.163749)100:0.212816,(cladig1.1_KAG5979913.1:0.148718,(clalov1.1_KAG5999390.1:0.0357206,clapus1.1_KAG5986315.1:0.0532833)100:0.110957)95:0.0441398)97:0.0283077)100:0.0868191,(((metacr1_6981:0.0555058,((metani1_9795:0.0143595,((metani2_KJK79258.1:2.2143e-06,metani3_KJK95579.1:2.2143e-06)100:0.00571308,methum1_KAH0593516.1:0.00860146)77:2.2414e-06)82:0.00571683,((metrob2.1_EXU97505.1:0,metrob4_mtdb2590:0)1:2.2143e-06,metrob3.1_EFY97960.1:2.2143e-06)100:0.0116881)96:0.00647723)87:0.0025186,(metbru1_4220:0.00558915,metgui1_9983:0.00846644)92:0.00304328)97:0.0437335,metalb1_1234:0.0639792)100:0.0776597)93:0.0614625,epifes1.1_QPG95279.1:0.151945)100:0.133987)85:0.025205,monsp.4_RYP93154.1:0.510986)84:0.028694,((asphan1.1_KAF7588053.1:0.0413591,asplep1_157608:0.0274096)100:0.215737,((asphom1_468681:0.0717019,penexp2_712:0.104877)100:0.105971,asplep1_20392:0.142184)99:0.0422449)84:0.0403759)98:0.0610199)56:0.0277212,((((dalchi1_KAF3063816.1:0.0753033,daldec1_30240:0.0565886)94:0.00299168,(dalloc2_490045:0.00566379,dalloc3_108807:0.0054325)99:0.0179215)100:0.143521,(triaru1_6994:0.0254096,tribre1_123775:0.0248111)100:0.103353)84:0.0221303,(xylnig1_360855:0.15617,xyltel1_247880:0.263826)100:0.0965652)52:0.0176456)50:0.00862311,monibe1_RYP10946.1:0.229596)77:0.0203132,psevol1.1_KAG9372089.1:0.130466)93:0.0157137,hypmon1_288472:0.0978274)88:0.0200155,((hypsp.10_85717:0.0265452,hypsp.8_352432:0.0366844)100:0.041429,hypsp.5_646361:0.0459435)100:0.0687836)99:0.0313574,(dalbam1_532614:0.0615451,((dalesc1_384250:0.00634326,(dalesc2_368012:2.2143e-06,dalsp.2_OTB14163.1:2.2143e-06)100:0.00810216)97:0.0149447,dalver1_236342:0.0397528)96:0.0150399)97:0.0491903);
