# Supplementary material for: Endophyte genomes support greater metabolic gene cluster diversity compared with non-endophytes in Trichoderma
Source: PLoS One. 2023 Dec 21;18(12):e0289280. doi: 10.1371/journal.pone.0289280 (PMC10735191; doi:10.1371/journal.pone.0289280)
Supplement: S12 Fig — Heat map showing presence/absence of biosynthetic gene cluster families for each Trichoderma genome. Singleton BGCs, as well as the highly fragmented T. cf. atroviride (LU140) genome, are included in this figure. The total number of BGC per genome, as well as the distribution of fungal-RiPP, other hybrid, and beta-lactone clusters, are unchanged from those in Fig 1 and are thus excluded for visualization purposes. Species lifestyle is indicated by presence/absence of color-coordinated cell, nutritional mode is indicated by either a circle (saprotroph) or a star (mycotroph). Endophyte cells half-filled indicated a potential endophytic lifestyle for that species. (PDF) [file pone.0289280.s012.pdf]

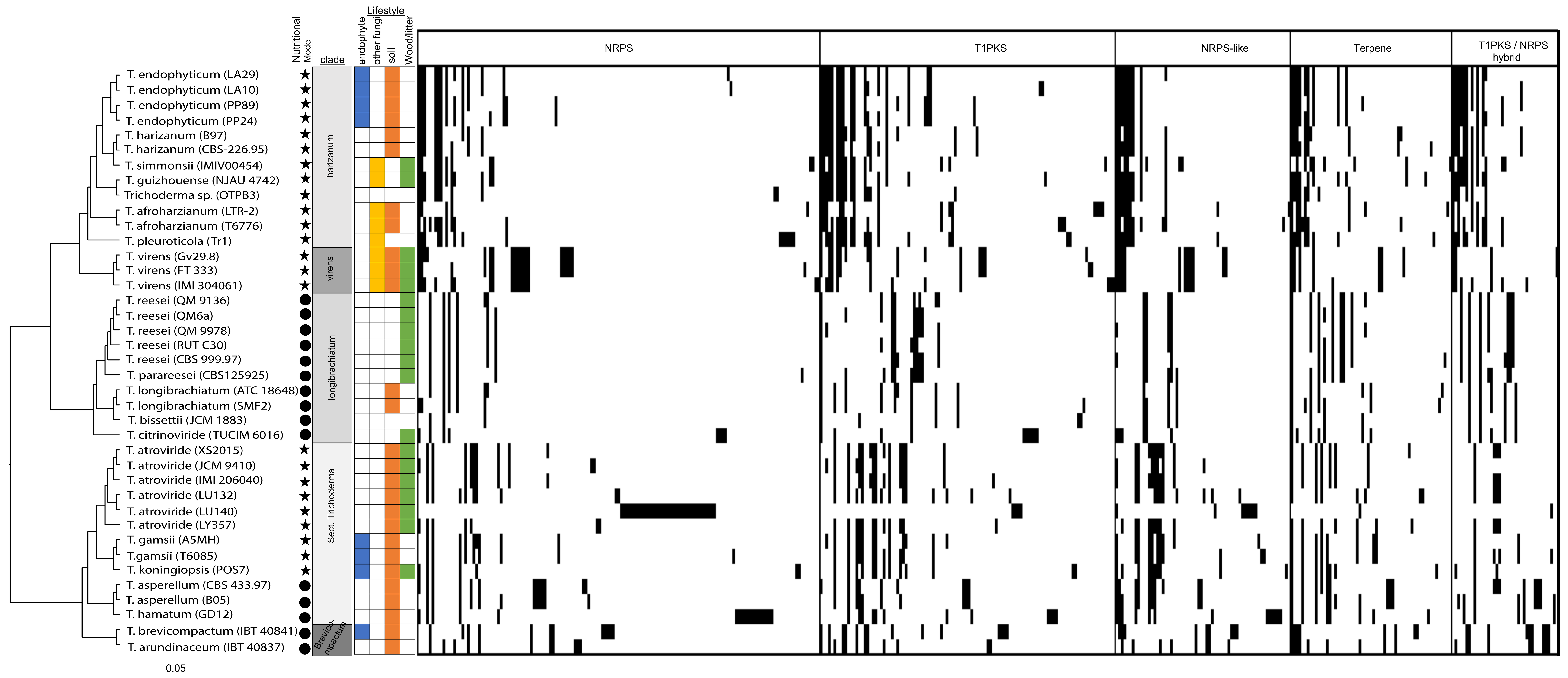

Nutritional Mode
clade
Lifestyle
endophyte
other fungi
soil
Wood/litter
NRPS
T1PKS
NRPS-like
Terpene
T1PKS / NRPS hybrid
T. endophyticum (LA29)
T. endophyticum (LA10)
T. endophyticum (PP89)
T. endophyticum (PP24)
T. harizanum (B97)
T. harizanum (CBS-226.95)
T. simmonsii (IMIV00454)
T. guizhouense (NJAU 4742)
Trichoderma sp. (OTPB3)
T. afroharzianum (LTR-2)
T. afroharzianum (T6776)
T. pleuroticola (Tr1)
T. virens (Gv29.8)
T. virens (FT 333)
T. virens (IMI 304061)
T. reesei (QM 9136)
T. reesei (QM6a)
T. reesei (QM 9978)
T. reesei (RUT C30)
T. reesei (CBS 999.97)
T. parareesei (CBS125925)
T. longibrachiatum (ATC 18648)
T. longibrachiatum (SMF2)
T. bissettii (JCM 1883)
T. citrinoviride (TUCIM 6016)
T. atroviride (XS2015)
T. atroviride (JCM 9410)
T. atroviride (IMI 206040)
T. atroviride (LU132)
T. atroviride (LU140)
T. atroviride (LY357)
T. gamsii (A5MH)
T.gamsii (T6085)
T. koningiopsis (POS7)
T. asperellum (CBS 433.97)
T. asperellum (B05)
T. hamatum (GD12)
T. brevicompactum (IBT 40841)
T. arundinaceum (IBT 40837)
harizanum
virens
longibrachiatum
Sect. Trichoderma
Brevicompactum
0.05
