# Supplementary material for: Endophyte genomes support greater metabolic gene cluster diversity compared with non-endophytes in Trichoderma
Source: PLoS One. 2023 Dec 21;18(12):e0289280. doi: 10.1371/journal.pone.0289280 (PMC10735191; doi:10.1371/journal.pone.0289280)
Supplement: Attachment — Submitted filename: Response to Reviewers_PONE-D-23-08909.docx [file pone.0289280.s037.docx]

**Responses to Reviewers' comments for Manuscript ID PONE-D-23-08909**

We thank the Reviewer 1 and the Academic Editor for their time and comments. Specific comments are addressed below and were also incorporated in the revised file. All edits made to the manuscript are visible under “track changes”.

**Reviewer 1’s comments:**

**Please define the terms mycotrophy and saprotrophy. For mycotrophy in particular, it was unclear to me how this term relates to endotrophy and mycoparasitism.**

Information has been added to the text as suggested (Lines 69-74). The introductory paragraph has been reworded to clarify the differences between lifestyle (endophyte/soil/wood/fungi) and nutritional mode (mycotroph aka mycoparasitism/saprotroph).

**I'd encourage the authors to read through the results section again carefully and add additional references throughout. Example statements that are missing attribution include lines 217, 221, 244, 884. References for Bloomberg's K, Pagel's Lambda, Fritz and Purvis' D-statistic are provided in the methods, but i think it would be helpful to include their references in the results section as well (lines 280, 293).**

References and comments have been added to the suggested lines.

**Can the author's perform a topology test to confirm that a constrained phylogeny in which Trichoderma easA genes group with other Hypocreales is significantly worse than the unconstrained best tree? I think this would be a nice additional bit of evidence to support the claim of HGT.**

Thank you for the recommendation to perform topology testing as we agree this would help support the inference of HGT. We conducted constraint analysis using the approximately unbiased topology tests (Table S7, referenced in revised text) and these indeed show topologies constrained to conform to vertical inheritance are significantly worse than the consensus tree.

**Additional comments regarding Editor’s edits/comments:**

As requested, permit information added to methods (Lines 901-904).

Grant info was removed from acknowledgements section.

Grant info was added to cover letter/online submission: National Science Foundation (DEB-1638999/DEB-1638976) to JCS and PC and National Science Foundation Grant (DEB-925672/DEB-1019972) to PC.

Abstract was amended so that the online submission form abstract and abstract in manuscript are identical.

The order of the reference list was changed due to the added requested citations, as well as the modified order of citations in the manuscript.

Additional formatting changes (header font size, case) were made to the manuscript to comply with the PLOS ONE format guidelines.
